# Mesenchymal stromal cell-derived extracellular vesicles afford neuroprotection by modulating PI3K/AKT pathway and calcium oscillations

**DOI:** 10.7150/ijbs.73747

**Published:** 2022-08-21

**Authors:** Egor A. Turovsky, Victoria V. Golovicheva, Elena G. Varlamova, Tatyana I. Danilina, Kirill V. Goryunov, Yulia A. Shevtsova, Irina B. Pevzner, Ljubava D. Zorova, Valentina A. Babenko, Ekaterina A. Evtushenko, Anastasia A. Zharikova, Anastasia A. Khutornenko, Sergey I. Kovalchuk, Egor Y. Plotnikov, Dmitry B. Zorov, Gennady T. Sukhikh, Denis N. Silachev

**Affiliations:** 1 Institute of Cell Biophysics of the Russian Academy of Sciences, Federal Research Center “Pushchino Scientific Center for Biological Research of the Russian Academy of Sciences”, 142290 Pushchino, Russia.; 2 A.N. Belozersky Institute of Physico-Chemical Biology, Lomonosov Moscow State University, Moscow 119992, Russia.; 3 Faculty of Biology, Lomonosov Moscow State University, Moscow 119234, Russia.; 4 V.I. Kulakov National Medical Research Center of Obstetrics, Gynecology and Perinatology, 117997 Moscow, Russia.; 5 Faculty of Bioengineering and Bioinformatics, Lomonosov Moscow State University, 119234 Moscow, Russia.; 6 Laboratory of Bioinformatics Methods in Combinatorial Chemistry and Biology, Shemyakin-Ovchinnikov Institute of Bioorganic Chemistry, Russian Academy of Sciences; 117997 Moscow, Russia.

**Keywords:** Multipotent mesenchymal stromal cells, extracellular vesicles, neuroprotection, neonatal hypoxic-ischemic encephalopathy, traumatic brain injury, calcium oscillations, the phosphatidylinositol 3‑kinase (PI3K)/protein kinase B (AKT) signaling pathway

## Abstract

Mesenchymal stromal cells (MSC) are widely recognized as potential effectors in neuroprotective therapy. The protective properties of MSC were considered to be associated with the secretion of extracellular vesicles (MSC-EV). We explored the effects of MSC-EV *in vivo* on models of traumatic and hypoxia-ischemia (HI) brain injury. Neuroprotective mechanisms triggered by MSC-EV were also studied *in vitro* using a primary neuroglial culture. Intranasal administration of MSC-EV reduced the volume of traumatic brain damage, correlating with a recovery of sensorimotor functions. Neonatal HI-induced brain damage was mitigated by the MSC-EV administration. This therapy also promoted the recovery of sensorimotor functions, implying enhanced neuroplasticity, and MSC-EV-induced growth of neurites *in vitro* supports this. In the *in vitro* ischemic model, MSC-EV prevented cell calcium (Ca^2+^) overload and subsequent cell death. In mixed neuroglial culture, MSC-EV induced inositol trisphosphate (IP3) receptor-related Ca^2+^ oscillations in astrocytes were associated with resistance to calcium overload not only in astrocytes but also in co-cultured neurons, demonstrating intercellular positive crosstalk between neural cells. This implies that phosphatidylinositol 3-Kinase/AKT signaling is one of the main pathways in MSC-EV-mediated protection of neural cells exposed to ischemic challenge. Components of this pathway were identified among the most enriched categories in the MSC-EV proteome.

## Introduction

According to the World Health Organization (WHO), the incidence of brain diseases accounts for one-third of the frequency of all diseases in developed countries 1. Neurological pathologies such as stroke, neonatal hypoxic-ischemic (HI) encephalopathy, and traumatic brain injury (TBI) are among the leading causes of mortality and disability 2,3. The development of therapeutic strategies for neuroprotection, aimed at improving cell survival and enhancing neuroplasticity in pathological conditions is of utmost importance 4. Despite the experimental confirmation of the effectiveness of various approaches to neuroprotection, its clinical implementation faces significant problems 5,6. The major obstacle in bench-to-bedside translation is the presence of multiple parallel signaling cascades leading to brain cell death, requiring the reconsideration of the use of unidirectional drugs.

While one way to solve this problem is the design of a multi-drug treatment, another one is transplantation of multipotent mesenchymal stromal cells (MSC), considered as a universal multi-purpose approach which has already demonstrated high efficacy towards acute brain pathologies 7-9. The positive effects of treatment with MSC is proposed to involve antiapoptotic activity, activation of angio- and neurogenesis, immunomodulation and activation of intercellular mitochondrial transport, which all might afford neuroprotection 10,11. A large body of experimental data indicates that the therapeutic effect of MSC is mediated by paracrine activity 12, rather than through the replacement of lost cells 13. The presence of neuroprotective elements in the secretome of stem cells is in support of the paracrine mechanism 14,15. Together with soluble substances, MSC-conditioned medium contains extracellular vesicles (EV), which can mimic the therapeutic effects of MSC (see for review 16), including neuroprotection 17. The composition of EV depends on the type of the parent cells, as well as on the challenges imposed on them, including the regimes of cultivation 18. They contain bioactive proteins, lipids and RNAs, including microRNAs, which play an important role in EV-mediated intercellular communication. There is a number of studies on proteomic profiling of EV produced by MSC (MSC-EV) 19-21, however, the causal relationship between the proteome and neuroprotection has not been explored. There are solitary studies demonstrating that EV contain some neuroprotective factors 22-24, however, a comprehensive understanding of the molecular mechanisms of therapeutic action of EV 25 is far from full understanding.

One of the important regulators of neuronal activity are calcium ions 26-29. However, calcium ions overload caused by acute brain injury has a remarkable pathogenic effect 30,31. It has been shown that under pathological conditions MSC can modulate intracellular calcium in neurons, however, the role of EV has not been explored 32.

The aim of this study was to test the hypothesis that MSC-EV afford neuroprotection through activation of signaling pathways to protect neural cells against calcium overload and, on the other hand, to activate the phosphatidylinositol 3-kinase/AKT (PI3K/AKT) pathway. In this regard, we studied the neuroprotective effects of MSC-EV in models of acute and hypoxic-ischemic brain damage, focusing on their ability to restore neurological functions within the long-term post-injury period. Molecular-cellular analysis of neuroprotective mechanisms activated by MSC-EV was supplemented by an *in vitro* study using primary neuroglial culture. Furthermore, we analyzed the protein and cytokine composition of MSC-EV in order to unravel possible elements of neuroprotective pathways activated by EV.

## Materials and methods

### Primary Culture of MSC

Healthy women of 22-26 years old (n = 8) were used as donors of postpartum placenta after successful delivery of healthy full-term infants by cesarean section at the V.I. Kulakov National Medical Research Center for Obstetrics, Gynecology, and Perinatology. No infectious diseases or pregnancy complications was in the medical history of these women as well as they were negative for hepatitis B virus (HBV), human immunodeficiency virus (HIV), and syphilis.

The inner part (approximately 1 cm^3^) of the central lobules of the placenta was washed in phosphate-buffered saline (PBS) (Paneco, Moscow, Russia) several times, cut into small pieces followed by their digestion by 100 U/ml collagenase type I (Gibco) in a serum-free Dulbecco's Modified Eagle Medium (DMEM) (Paneco, Moscow, Russia). Obtained cell suspensions were collected by centrifugation for 5 min at 300 × *g,* washed with DMEM and spinned again at 300 *g* for 5 min. The cells were suspended in DMEM/F12 (Paneco, Moscow, Russia) (1:1) containing 7% fetal bovine serum (FBS) (Biosera, Nuaille, France) supplemented with penicillin (100 IU/mL), streptomycin (100 μg/mL) (Gibco, NY, USA), and 2 mM L-glutamine (Paneco, Moscow, Russia). Prior to use, the cultivation medium was spinned at 108,000 × g for 1.5 h at 4 °C in an Avanti JXN-30 centrifuge (Beckman Coulter Inc., Fullerton, CA, USA) to avoid possible contamination with EV derived from FBS. The supernatant was harvested, filtered using a bottle-top vacuum filter system with a pore size of 0.22 μm (Falcon, Corning, NY, USA), and used for further experiments. Cells were transferred into a single 75 cm^2^ tissue culture flasks (Gibco Life Technologies, Baltimore, MD, USA) and incubated in a humidified atmosphere with 5% CO_2_ at 37 °C. The cultivated medium was changed every 3-4 days to remove nonadherent cells. Cell growth and morphology were visually monitored daily. MSC at the third to fourth passage were used for harvesting extracellular vesicles in three-layer flasks (Nunc, Thermo Fisher Scientific, Dreieich, Germany).

### Isolation of Extracellular Vesicles by Differential Centrifugation

EV were isolated from MSC-cultured media following the guideline recommended by International Society for Extracellular Vesicles (ISEV), called Minimal Information for Studies of Extracellular Vesicles 2018 (MISEV 2018) 33.

Differential centrifugation was used in a procedure to isolate EV as described 34. Briefly, conditioned medium (50 mL) from confluent cultures at 80-90% confluence (~25×10^6^ cells) was collected 24 h after medium refreshment and exposed to serial centrifugations to remove cells and debris (400 × g for 10 min followed by 10,000 × g at 4 °C for 30 min). Supernatant was used for EV isolation by repetitive ultracentrifugation at 108,000 × g for 1.5 h at 4 °C and wash with PBS to minimize protein contamination. The final EV pellet was resuspended in 30 μL of PBS for experiments *in vitro* and *in vivo* and 1 ml for nanoparticle tracking analysis (NTA). Vesicles were stored at -80 °С. A resuspended pellet from nonconditioned cultivation medium exposed to identical series of centrifugations was used as a control sample (blank EV) to ensure that the observed effects were caused by EV from MSC rather than by an unavoidable admixture of adventitious nanoparticles. The EV proteins were quantified using the Pierce BCA Protein Assay Kit (Thermo Scientific, Rockford, IL, USA) according to the manufacturer's instructions.

### Analysis of the size of extracellular vesicles

Size distribution of vesicles was measured using a NanoSight LM10 (Malvern Panalytical, London, UK) equipped with blue laser (405 nm, 60 mW) and CMOS camera (Hamamatsu Photonics K.K., Hamamatsu City, Japan). An EV pellet resuspended in 1 ml PBS was diluted 10,000 times by serial dilution with PBS to reach the optimal concentration for instrument linearity (20-30 particles/frame), and readings were performed in triplicates of 30 s at 30 frames per second. For analysis of concentration and size-distribution, 12 exposures of 60 seconds each were recorded with 15-22 particles per image. Data were analyzed with NTA 2.3 software using the following settings: calibration - 166 nm/pixel; blur - auto; detection threshold - 8, minimum track length - auto, minimum expected particle size - 30 nm, temperature - 24.7 °C, viscosity - 0.90 cP. Calibration was performed using Nanosight size transfer standards (100, 200, 400 nm) and Nanosight fluorescence standards (Malvern Panalytical, UK).

### Transmission Electron Microscopy

A 10 μL drop of EV in PBS was applied to nitrocellulose carbon-coated PELCO® Cu grids (Ted Pella Inc., Redding, CA, USA) and incubated for 1 min. Liquid was removed by touching the top of the grid with filter paper. This grid was exposed to a 10 μL drop of 2% uranyl acetate, followed by 10 s incubation with further removal of the moisture by touching the grid with the filter paper. Samples were examined in a transmission electron microscope, JEOL JEM-1400 TEM (JEOL, Tokyo, Japan) attached to an Olympus Quemesa digital camera and analyzed by iTEM software (Olympus Soft Imaging Solutions GmbH, Munster, Germany).

### TBI Model and treatment with EV

The 4-months old outbred Wistar male rats (350-400 g) used in the study were raised in the animal facility of the A.N. Belozersky Institute of Physico-Chemical Biology. The rats had unlimited access to food and water and were kept in a temperature-controlled cages (20±2 °C) with light on from 9:00 a.m. to 9:00 p.m. In all surgical experiments, rats were anesthetized with isoflurane (2.5% induction, 1.5% maintenance) using a rodent anesthesia vaporizer (VetFlo, Kent Scientific Corporation, Torrington, CT, USA). To provide proper pain relief in the perioperative and postoperative periods, a repeated topical application of bupivacaine ointment, was employed. A feedback-controlled heating pad maintained the core temperature (37.0±0.5 °C) and was supplemented with an infrared lamp during surgery and until awake.

**Figure 1:**
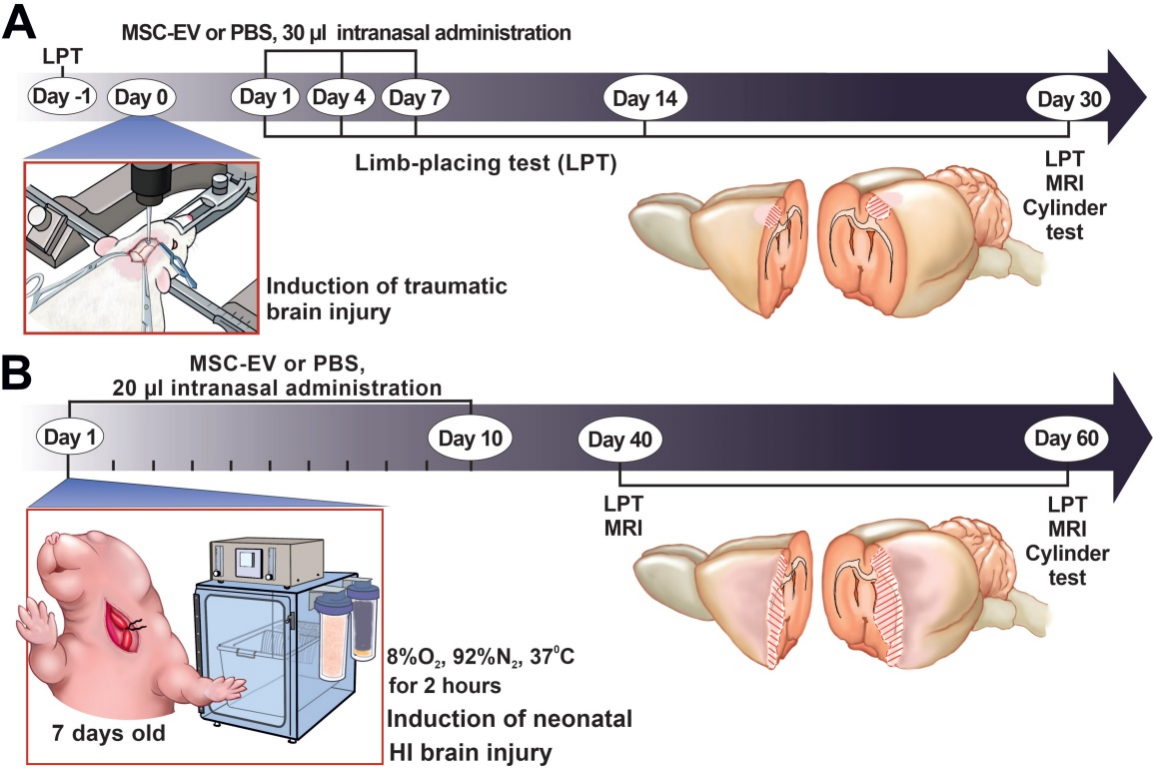
** The study design. (A)** Scheme of the experiment for studying the neuroprotective properties of MSC-EV in the TBI model. **(B)** Scheme of the experiment for studying the neuroprotective properties of MSC-EV in the neonatal HI model. LPT - limb-placing test, MRI - magnetic resonance imaging.

In the study, we employed our modification of an earlier described model of focal open brain trauma in rats 35,36. To generate the trauma, the rats were positioned in a stereotaxic frame (NeuroStar Robot Stereotaxic, Germany), the right frontal part of the skull was trepanned above the sensorimotor cortex zone and a movable Teflon piston 4 mm in diameter and 50 g weight was dropped on it from the height of 10 cm sliding along a directing rail ultimately penetrating into the depth of insertion of 2.5 mm. For localization of the sensorimotor cortex zone, we used the following stereotaxic coordinates: +4 to -3 mm anterior and posterior from bregma and +1 to +4.5 mm lateral from the midline. In sham-operated rats, the experiments were done using the same protocol except that trauma was excluded. Rats were randomly divided into three groups according to surgery and subsequent intranasal (i/n) instillation: (1) Sham+PBS (n=7); (2) TBI+PBS (n=14); (3) TBI+MSC-EV (n=10). Thirty microliters of PBS or MSC-EV resuspended solution containing 1.6 ± 0.2 × 10^11^ particles/mL of EV was administered intranasally as drops released from a fine tip every 5 min into both nostrils, followed by 5 μL for the last dose (for a total of 20 min). Rats were treated with 30 μL of EV suspension on the 1^st^, 4^th^ and 7^th^ days after TBI induction. The volume of damage was evaluated after analyses of the brain magnetic resonance (MR) images obtained 30 days in the post TBI period (see MRI protocol below). Neurological status was determined with the limb-placing test before TBI (baseline) and 1, 4, 7, 14 and 30 days after TBI (see below for details). The cylinder test (see below) was used to assess forelimb use asymmetry at 30 days after TBI (Figure 1A).

### Induction of neonatal HI brain injury and treatment with EV

Dams and their pups were kept in cages under a constant temperature (20 ± 2 °C) with light on from 9:00 a.m. to 9:00 p.m. Dams had ad libitum access to food and water, and pups were checked daily for health. For a long-term survival and behavioral testing, the pups were weaned on P30 after HI induction, and 5-6 animals per cage were housed (split by sex) with the later testing for neurological function.

In the study, the modified Rice-Vannucci rat model of HI brain injury 37 was used. Seven-day-old rat pups underwent permanent unilateral carotid ligation under isoflurane anesthesia (2.5% induction, 1.5% maintenance) followed by 120-min exposure to 8% oxygen at 35 °C. The mortality rate after the HI induction procedure was 3-5%. Pups from at least six litters were randomly divided into the following experimental groups: HI + PBS (n = 12), pups with HI induction that received i/n instillation of PBS; HI + MSC-EV (n = 11), pups with HI treated by i/n instillation with 20 µL MSC-EV suspension containing 1.6 ± 0.2 x 10^11^ particles/mL of MSC-EV. Pups were treated with EV daily for two weeks, excluding weekends, for a total of 10 treatments (Figure 1B). The first intranasal delivery of EV was conducted 1 hour after HI induction. Pups were gently placed on their backs and 20 µL MSC-EV suspension were divided on 2 μL solution for instillation every 5 min into each naris using a fine tip and the pups were returned to their dams. Control groups of animals received an equal volume of PBS by the same treatment scheme. The extent of damage was quantified by analyzing brain MR images obtained 40 and 60 days after HI induction. Neurological status was determined with the limb-placing test at 40 and 60 days and the cylinder test (see below) at 60 days after HI induction.

### MRI studies of brain damage

Infarct volume was quantified by analyzing brain MRI images as described previously 38 on a 7T scanner (Bruker BioSpec 70/30 USR, Bruker BioSpin, Ettlingen, Germany) using 86 mm volume resonator for radio frequency transmission and a phased array rat head surface coil for reception. Before scanning, the animals were anesthetized with isoflurane (2% induction, 1.5% maintenance) in a mixture of oxygen and air. Rats were placed in a prone position on a water-heated bed. The head of the rat was immobilized using a nose mask and masking tape. The imaging protocol included a T2-weighted imaging sequence (repetition time = 4500 ms; echo time = 12 ms; slice thickness = 0.8 mm). Ischemic damage volume was outlined using ImageJ software (NIH, Bethesda, MD, USA).

### Limb-placing test

The modified version of the limb-placing test consisting of seven tasks was used to assess forelimb and hindlimb responses to tactile and proprioceptive stimulation 39. Rats were handled three days before testing. For each task, the following scores were used: 2 points, normal response; 1 point, delayed and/or incomplete response; 0 points, no response. The mean score was evaluated over seven tasks.

### Cylinder test

Asymmetry of forelimb use was evaluated in a cylinder test based on spontaneous exploration of the cylinder walls 40. The rat was placed into a transparent cylinder (30 cm height and 20 cm in diameter) and its movements were recorded over 5-8 min with a camcorder positioned above the cylinder. The independent use of the contra- and ipsilateral forelimbs during cylinder wall exploration in a rear posture and their simultaneous (combined) use were counted. The frequency of forelimb use was calculated by the formula 100×(*contr*+1/2×*simult*)/(*ipsi*+*simult*+*contr*), where *contr* and *ipsi* corresponded to the use of contralateral (impaired) and ipsilateral limbs and *simult* corresponded to simultaneous use of both forelimbs.

### Hippocampal cell culture

A mixed neuroglial culture of hippocampal cells was obtained from newborn (P1-3) Spraque Dawley rats. After decapitation, the hippocampus was extracted and transferred to a cold Hanks' balanced salt solution (HBSS). The tissue was crushed with scissors, placed in an EDTA solution with the addition of 0.2% trypsin and incubated for 10 min at 37 °C on a thermoshaker at 600 rpm. The pieces of tissue treated with the enzyme were washed three times with a neurobasal medium (Gibco, Waltham, MA, USA), carefully pipetted and centrifuged (2 min at 300×g). The supernatant was then removed, and the cells were resuspended in a neurobasal medium with the addition of glutamine (0.5 mM), supplement B27 (2%) and gentamicin (15 µg/ml) (all Gibco, Waltham, MA, USA). The suspension was introduced into glass cylinders with polished ends with an internal diameter of 6 mm, standing on round cover glasses with a diameter of 25 mm (VWR International) coated with polyethylenimine, and placed in 35 mm Petri dishes (Greiner). 100 µl of cell suspension was added to each cylinder and left for 2 hours to attach in a CO_2_ incubator at 37 °C. After that, the cylinders were removed, and the volume of the culture medium was adjusted to 1.8 ml. Every 3 days, 2/3 of the volume of the culture medium was replaced with fresh media. The experiments were carried out using cultures of 12-15 days *in vitro* (DIV).

### Modeling of oxygen-glucose deprivation

Ischemia-like conditions (oxygen-glucose deprivation, OGD) were obtained by omitting glucose (HBSS medium without glucose) and by displacement of dissolved oxygen with argon in the leak-proof system as described earlier (41). Partial oxygen tension was lowered until 30-40 mm Hg (as measured with a Clark-type oxygen electrode) corresponding to acute hypoxia. In turn, to model ischemia-like conditions, glucose was substituted by an equivalent quantity of sucrose (HBSS without glucose) for 40 min. To prevent contact of the medium with ambient O_2_, the experimental chamber was continuously flushed with argon. The neuroglial culture was pretreated with MSC-EV at a concentration of 2.4 × 10^8^ particles/mL 24 hours before OGD induction. The effect of OGD on cells was assessed by the amplitude and mode of Ca^2+^ signals, and necrotic cells were counted using the propidium iodide (PI) staining approach.

### A model of hyperammonemia

Primary hippocampal cell cultures were obtained from a single rat and after 10 days of cultivation, they were divided into 3 groups. The cells were washed with PBS and treated with two different concentrations of MSC-EV (2.4 × 10^8^ and 1.6 × 10^8^ particles/mL) for 24 hours. The cells of the third group were treated with an equivalent volume of HBSS. After 24 hours, 8 mM NH_4_Cl (hyperammonemia model) was added to Petri dishes with cells and kept for 48 hours, after which the cells were loaded with Propidium iodide and Hoechst-33342 and the fluorescence from 3 arbitrarily chosen regions was measured for each Petri dish. The ratio of living cells and cells with necrosis and apoptosis was determined.

### Fluorescent Ca^2+^ measurements

The concentration of calcium ions in the cytoplasm ([Ca^2+^]_i_) was evaluated using the two-wavelength probe Fura-2 AM (Thermo Fisher Scientific, Waltham, MA, USA) in accordance with the manufacturer's instructions. To stain hippocampal cells, Fura-2 AM at a final concentration of 4 µM in a Hanks' solution was used containing (in mM): 156 NaCl, 3 KCl, 1 MgSO_4_, 1.25 KH_2_PO_4_, 2 CaCl_2_, 10 glucose and 10 HEPES, pH 7.4. Freshly prepared dye solution (200 µl) was added to the coverslip with hippocampal cells and incubated for 40 min at 37 °C. The coverslip was washed with a HBSS and incubated for 10-15 min to let the completion of deesterification of the dye.

Changes in the levels of Ca^2+^ in the cytoplasm of cells were analyzed by the Cell Observer image analysis system (Carl Zeiss, Germany) based on an Axiovert 200M inverted microscope attached to AxioCam HSm CCD camera with a Ludl MAC5000 high-speed wheel containing excitation filters. A Plan-Neofluar 10×/0.3 lens was used to visualize the object. Fluorescence was excited by a HBO 103W/2 mercury lamp with proper excitation filters including a 21HE filter set (Carl Zeiss, Germany) consisting of BP340/30 and BP387/15 excitation filters, FT409 beam splitter and BP510/90 emission filter to record Fura-2 fluorescence. To measure the fluorescence, a round coverslip with a cell culture on it was mounted in a special measuring chamber. The volume of the medium in the chamber was 0.5 ml. Reagents were added and washed by replacing the medium by a tenfold volume using a system that provided perfusion at a rate of 15 ml/min. The measurements were carried out at 28 °C. A series of images was obtained with an interval of 1 frame per 3 seconds. The identification of neurons was carried out by a short-term addition of 35 mM KCl, which causes depolarization and an increase in the concentration of cytosolic calcium in excitable cells (in this case, neurons). The reaction of Ca^2+^ in neurons in response to the use of high concentrations of K^+^ is characterized by rapid growth and slow decline. This response is absent in glial cells 42,43.

The time series of two-channel images (at 340 and 380 nm excitation) were processed in the ImageJ program with the Time Series Analyzer software module. The amplitude of the calcium responses of single cells was measured as the ratio of Fura-2 fluorescence signals with excitation at the two wavelengths. The area under the curve is constructed in Origin 8.5 using the Polygon Area function for curves averaged over several dozen cells. The results are presented as signals received from individual cells inherent for neurons and astrocytes, or as an average signal taken from cells in the field of view ± standard error (SE).

### Cell survival analysis

The number of dead cells in the same culture before and after OGD was determined by staining cultures with 1 µM PI. The viability of cells was also assessed from the shape of the calcium signal under OGD (as the presence or absence of a global increase in [Ca^2+^]_i_ during OGD).

The effect of MSC-EV on NH_4_Cl-induced activation of apoptosis and necrosis was analyzed by simultaneous staining of cells with PI and Hoechst 33342. The approach is based on the inability of *viable* cells to be stained with PI, while Hoechst 33342 stains chromatin in *all* cells. According to a commonly used method 44,45, hippocampal neurons were defined as apoptotic when the intensity of Hoechst fluorescence was 3-4 times higher compared to the fluorescence in healthy cells, indicating chromatin condensation caused by apoptosis induction. The fluorescence of the probes was recorded with a fluorescent system based on an Axio Observer Z1 inverted fluorescent microscope equipped with a Hamamatsu ORCA-Flash 2.8 high-speed monochrome CCD-camera. The Lambda DG-4 Plus illuminator (Sutter Instruments, USA) was used as a source of excitation of fluorescence. To excite and record fluorescence of the probes, the Filter Set 01 with excitation filter BP 365/12, beam splitter FT395 and emission filter LP 397 or Filter Set 20 with excitation filter BP 546/12, beam splitter FT560 and emission filter BP 575-640 were used. Five different fields were examined for each coverslip.

### Monitoring cell morphology and neurite growth

The primary neuroglial culture was obtained as described above and divided into two groups 4 h after the cells were attached. First group was treated by MSC-EV at a concentration of 2.4 × 10^8^ particles/mL, while the control group was treated with vehicle and the culture medium was not changed during the entire experiment. The analysis of cell cultures was carried out daily for 2 plates from each group during 7 days, after which they were not used. Hippocampal cell cultures were loaded with a Calcein-AM probe at a final concentration of 5 µM for 40 min at 37 °C in HBSS with 10 mM HEPES followed by a triple wash with HBSS. Cell slips were mounted in an experimental chamber and observed using a Leica DMI6000B fluorescent inverted motorized microscope with a HAMAMATSU C9100 high-speed monochrome CCD camera. For excitation and recording of Calcein fluorescence, a L5 filter set (Leica, Germany) was used, containing excitation filter BP480/40, beam splitter FT-505 and emission filter BP527/30, with a Leica EL6000 excitation light source containing a HBO 103 W/2 high-pressure mercury lamp. Neurite length analysis was performed using the Image J software.

### Western blot analysis

Samples of cell lysates and EV were loaded onto gradient (5-20%) Tris-glycine polyacrylamide gels (10 µg of total protein per lane for cell lysates and 0.3 µg of total protein per lane for EV). After electrophoresis, gels were blotted onto PVDF membranes (Amersham Pharmacia Biotech, Newcastle, UK). Membranes were blocked with 5% (wt/vol) non-fat milk (SERVA, Germany) in PBS with 0.05% (vol/vol) Tween 20 (Panreac, Barcelona, Spain) and subsequently incubated with primary antibodies: rabbit polyclonal anti-CD9 1:1000 (#HBM-CD9, HansaBioMed, Tallinn, Estonia), rabbit polyclonal anti-CD81 1:1000 (#HBM-CD81, HansaBioMed, Tallinn, Estonia), rabbit polyclonal anti-TSG101 1:1000 (Tumor susceptibility gene 101, #M01233, BOSTER Biological Technology, Pleasanton, CA, USA), mouse monoclonal anti-Vimentin 1:1000 (#V9, Cell Marque, Rocklin, USA), mouse monoclonal anti-cytochrome C 1:2000 (#556433, BD Pharmingen, San Jose, CA, USA). Membranes were stained with secondary antibodies anti-rabbit IgG or anti-mouse IgG conjugated with horseradish peroxidase 1:5000. Specific bands were visualized using an Advansta Western Bright™ ECL kit (Advansta, San Jose, CA, USA). Detection was performed with a V3 Western Blot Imager (BioRad, Hercules, CA, USA) and the density of the bands was measured using Image Lab software (BioRad, Hercules, CA, USA). Protein concentration was measured by bicinchoninic acid assay (Sigma, St. Louis, MO, USA).

### Sample preparation for proteomics

EV were lysed in a buffer containing 1% sodium deoxycholate (SDC), 100 mM TRIS, pH 8.5 with MS-SAFE protease inhibitor cocktail (Sigma-Aldrich) by ultrasonication with a QSonica probe sonicator at 4 °C. Protein concentration was estimated by microBCA (ThermoScientific). Aliquots containing 50 µg of total proteinl were diluted to 1 mg/ml with the lysis buffer and tris(2-carboxyethyl)phosphine (TCEP) and 2-chloroacetamide (CAA) were added to reach the concentrations of 10 and 20 mM respectively. Cys reduction and alkylation was achieved by 10 min heating of the sample at 80 °C. Proteins were precipitated by addition of a four-fold volume of acetone and incubation at -20 °C overnight and spinned down in the centrifuge. The protein pellet was washed twice with acetone. The pellet was then resuspended in 50 µl of the lysis buffer in a sonication bath. Trypsin (Promega, USA) was added at the ratio 1/100 w/w to the protein content and the mixture was incubated for 2 hours at 37 °C followed by the second trypsin treatment of 1/100 w/w and left overnight at 37 °C. Proteolysis was stopped by adding trifluoroacetic acid (TFA) to 1% v/v. Precipitated SDC was removed by centrifugation. The samples were loaded onto the LC-MS instrument directly without solid phase extraction (SPE).

### Protein Mass Spectrometry

LC-MS analysis was carried out using an UltiMate 3000 RSLCnano HPLC system connected to a QExactive Plus mass spectrometer (ThermoFisher Scientific). Samples were loaded to a home-made 20×0.1 mm trap column, packed with Inertsil ODS3 3 µm sorbent (GLSciences), in the loading buffer (2% acetonitrile (ACN), 98% H_2_O, 0.1% TFA) at 10 µl/min flow and separated at room temperature in a home-made 500×0.1 mm fused-silica column packed with Reprosil PUR C18AQ 1.9 (Dr. Maisch Gmbh, Germany) into the emitter prepared with P2000 Laser Puller (Sutter, USA) 46. Samples were eluted with a linear gradient of 80% ACN, 19.9% H_2_O, 0.1% formic acid (FA) (buffer B) in 99.9% H_2_O, 0.1% FA (solvent A) from 4 to 36% of solvent B in 1 h at a flow rate of 0.44 µl/min at RT.

MS data were collected in DDA mode. MS1 parameters were as follows: 70K resolution, 350-2000 scan range, max injection time 50 ms, AGC target 3×10^6^. Ions were isolated with 1.4 m/z window and 0.2 m/z offset targeting the 10 highest intensity peaks of +2 to +6 charge, 8×10^3^ minimum AGC, preferred peptide match and isotope exclusion. Dynamic exclusion was set to 40 s. MS2 fragmentation was carried out in HCD mode at 17.5K resolution with 27% NCE. Ions were accumulated for a maximum of 45 ms with a target AGC of 1×10^5^. Each sample was analyzed in 2 technical repeats.

### Proteomic data analysis

Raw spectra were processed in MaxQuant 1.6.6.0 (MQ) 47 and Perseus 48. The data was searched against the Human Uniprot SwissProt database, containing canonical proteins, version 2019/10/03.

The MaxQuant search was performed with the default parameter set, including Trypsin/p protease specificity, a maximum of 2 missed cleavages, Met oxidation, Protein N-terminal acetylation and NQ deamidation as variable modifications and Carbamidomethyl Cys as a fixed modification with a maximum of 5 modifications per peptide, 1% PSM and protein FDR. The following options were turned on: second peptide, maxLFQ, match between runs. All runs were analyzed as independent experiments and processed in Perseus.

In Perseus, the protein group results were filtered for contaminants, reverse and “identified only by site” proteins. Only the proteins with maxLFQ values in at least 4 out of 8 LC-MS runs were used. For them, missing values were imputed from a normal distribution with 0.3 intensity distribution sigma width and 1.8 intensity distribution center downshift.

The mass spectrometry proteomics data have been deposited to the ProteomeXchange Consortium via the PRIDE 49 partner repository with the dataset identifier PXD027581.

### Bioinformatic analysis

Functional annotation of MSC-EV proteins was performed using DAVID Bioinformatics Resources, version 6.8 50,51, and the Gene Ontology resource (release date - 2021-09-01; version number - 10.5281/zenodo.5395002) (Supplementary Table 1) 52,53.

### Cytokine multiplex assay

The cytokines and chemokines in the MSC-EV were analyzed using the Bio-Plex Pro™ Human Cytokine Screening Kit, 48-Plex according to the manufacturer's protocol (Bio-Rad, Munich, Germany). Samples were measured in duplicates using the Bio-Plex^®^ 200 system (Bio-Rad, Munich, Germany). Cytokine concentrations were determined with the Bio-Plex^®^ Manager Version 6.0 (Bio-Rad, Munich, Germany).

### Statistics

Data were tabulated and statistical analysis and graphs were generated with MS Excel 2010 (Microsoft Corporation Redmond, Washington, U.S.), Origin 2016 (OriginLab, Northampton, MA, USA), and Prism GraphPad 7 (GraphPad Software, San Diego, CA, USA). Normality of distribution was assessed by Shapiro-Wilk test. All values are given as mean ± standard error (SEM). Statistical analyses were performed by one-way ANOVA followed by Tukey's post hoc test or two-way ANOVA followed by Sidak's or Tukey-Kramer multiple comparison test or by paired t-test. For cell culture experiments, all presented data were obtained from at least three cell cultures from 2-3 different passages. Differences with a probability (p) value less than 0.05 were considered statistically significant.

## Results

### Characterization of MSC-derived EV

**Figure 2:**
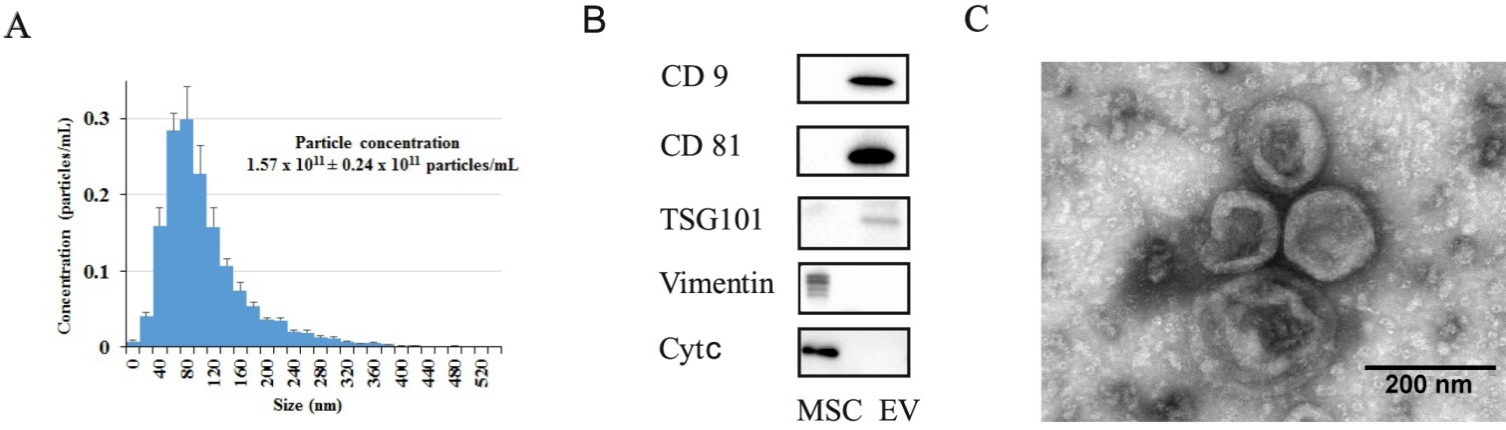
** Characterization of MSC-derived EV obtained from conditioned medium after 24 h of MSC cultivation. (A)** Particle size distribution of EV preparation. Shown are the means ± SEM for 3 independent cell cultures. **(B)** Detection of EV markers (CD9, CD81, TSG101) and non-EV markers (vimentin, cytochrome c) by Western blot analysis. **(C)** Transmission electron microscopy of EV preparation.

EV were characterized in terms of particle concentration, morphology, surface and protein markers. NTA revealed that they were relatively homogenous particles with size distribution 20-360 nm (peak of 80 nm) and mean particle diameter 110±9.3 nm. The total particle concentration in a sample of EV isolated from MSC-cultured media was 1.57±0.24 × 10^11^ /ml (Figure 2A). SDS-PAGE electrophoresis and Western blotting revealed that EV contained conventional EV markers including СD9, CD81 and TSG101 and did not contain a detectible amount of vimentin or cytochrome *c*, which were detected in MSC (Figure 2B). TEM of EV attached to nitrocellulose carbon-coated grids (Figure 2C) confirmed that most objects had a cup-shape morphology characteristic of EV, with sizes ranging from 40 to 250 nm. Proteomic analysis revealed that EV were positive for most EV markers recommended by MISEV2018 33. Moreover, proteomic analysis detected 91 of the top 100 most identified exosomal marker proteins from the ExoCarta database (54-56) (Supplementary Table 1 - Exocarta top100). As a result of DAVID functional annotation, 552 proteins (out of 745, i.e., 74%) were related to the term 'extracellular exosome' (Supplementary Table 1 - DAVID functional annotation).

### MSC-EV protect rat brain exposed to TBI

**Figure 3:**
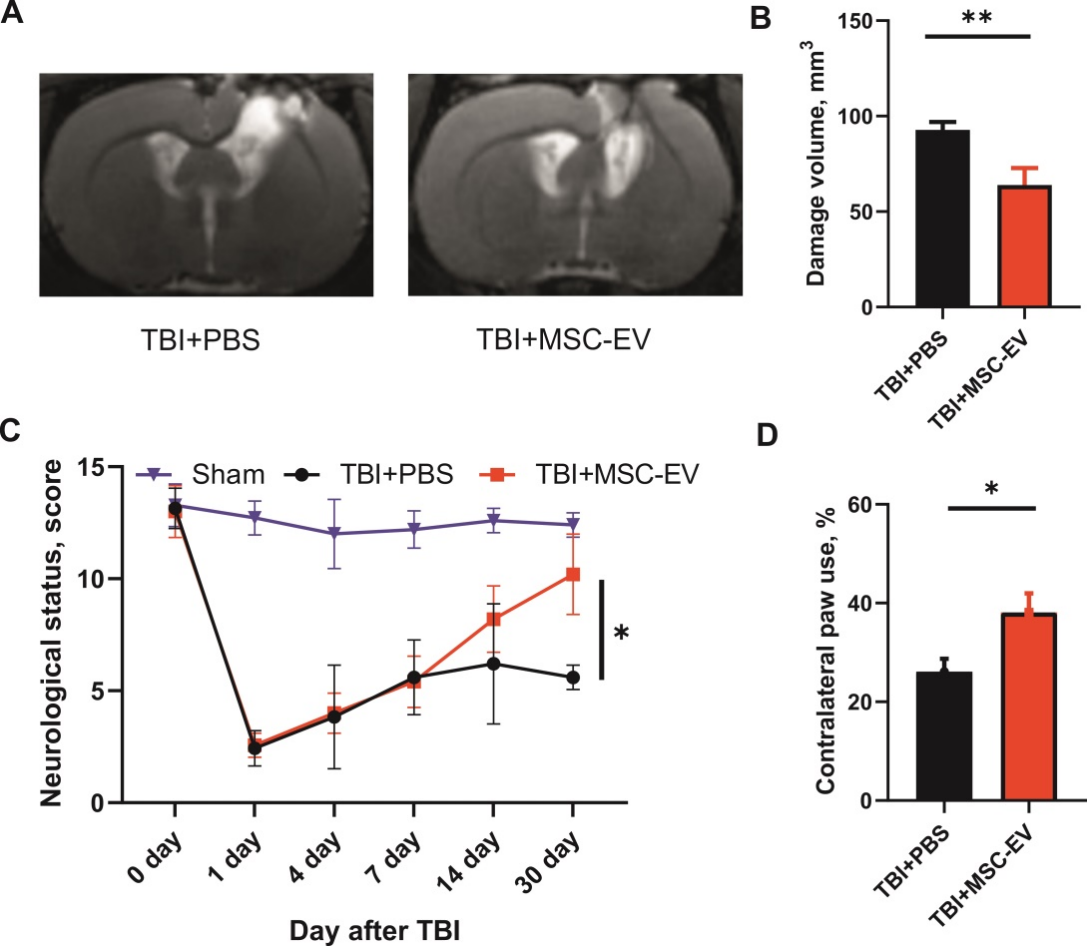
** MSC-EV treatment protects against TBI. (A)** Representative T2-weighed MR images of coronal brain sections taken 30 days after TBI induction; **(B)** Damage volume evaluated by MRI; **p=0.0037, unpaired t-test. **(C)** Effect of MSC-EV on neurological status determined by a limb-placing test up to 30 days after TBI; *p=0.0106, two-way ANOVA, post-hoc Sidak's test in comparison with untreated TBI group. **(D)** Effect of MSC-EV on asymmetry in forepaw use evaluated in the cylinder test 30 days after TBI. *p=0.0109, unpaired t-test compared with untreated TBI group. Data are shown as means ± SEM. TBI+PBS, animals treated with 30 µl PBS (n=14); TBI+MSC-EV - animals treated with 30 µl MSC-EV on 1st, 4th, and 7th days after injury (n=10); sham-operated animals (n=7).

Analysis of MR images obtained on day 30 after TBI revealed significant damage to the sensorimotor cortex (Figure 3A). The volume of brain lesion in control animals receiving PBS alone was 92.8±4.1 mm^3^. Intranasal administration of MSC-EV on days 1, 4, and 7 after induction of TBI resulted in significantly smaller lesion volume, reaching 63.9 ± 8.8 mm^3^ (p = 0.0037; Figure 3B).

Damage to the sensorimotor cortex led to pronounced neurological disturbances of the paws on the contralateral side. Specifically, the results of the limb-placing test demonstrated significant sensorimotor deficits in the left limbs caused by TBI. While the intact rats before the induction of TBI scored 14.0±0 in this test and sham-operated animals scored 12.7±0.8, rats treated with PBS demonstrated only 5.6±0.3 points on day 30 after TBI. The treatment with MSC-EV significantly restored the neurological status to 10.2±0.5 points (p=0.0106; Figure 3C) on day 30 after TBI. Using the cylinder test, we found that TBI results in an asymmetry in the use of the forelimbs. Normally, rats use both the left and right forelimbs in the same proportion when exploring the walls of the closed space in the glass cylinder test. Quantitative analysis showed that 30 days after the induction of TBI, the frequency of the contralateral paw usage was only 26.1 ± 2.6% in the TBI+PBS group. TBI rats subjected to intranasal instillation of MSC-EV exhibited a significantly greater contralateral forepaw use of 38.1 ± 3.8% (p =0.0109; Figure 3D).

### MSC-EV protect neonatal brain against HI

**Figure 4:**
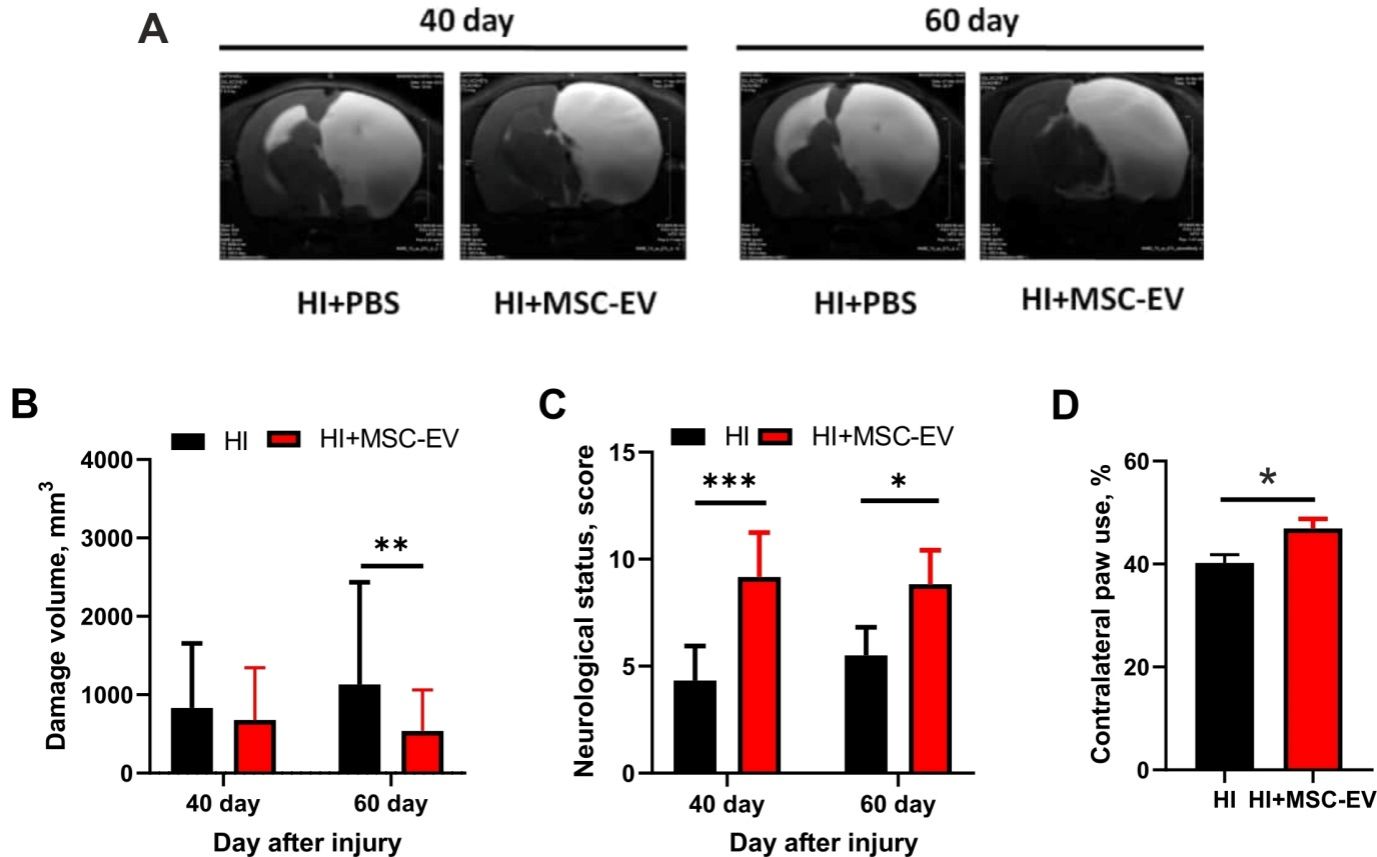
** Evaluation of the neuroprotective effect of MSC-EV 40 and 60 days after induction of brain HI injury in rat pups. (A)** Representative T2-weighed MR images of coronal brain sections on 40 and 60 days after HI induction. Hyperintense regions in the right hemisphere are identified as ischemic areas; **(B)** Infarct volume evaluated by analyzing T2-weighted images; **p<0.01, two-way ANOVA, post-hoc Sidak's test comparing EV-treated HI group (shown as HI+MSC-EV) with HI+PBS group (shown as HI). **(C)** Effect of MSC-EV on neurological status determined by a limb-placing test 40 and 60 days after HI. *p<0.05, ***p<0.0001, two-way ANOVA, post-hoc Sidak's test comparing the HI+MSC-EV and HI+PBS groups. **(D)** Effect of MSC-EV on asymmetry in forepaw use evaluated in the cylinder test 60 days after induction of HI injury. *p<0.05, unpaired t-test comparing HI+MSC-EV and HI+PBS groups. Data are shown as means ± SEM. HI - animals treated with 20 µl PBS during 10 days after injury (n=12); HI+MSC-EV - animals treated with 20 µl MSC-EV during 10 days after injury (n=11).

We examined HI-induced damage in the sensorimotor cortex using MRI on the 40^th^ and 60^th^ days after induction of HI injury (Figure 4A). HI caused a remarkable damage to the brain, occupying approximately 85% of the volume of the affected hemisphere 40 days after HI. We found no statistically significant difference in lesion volume between the HI+PBS and HI+MSC-EV groups 40 days after induction of HI injury. However, we observed subsequent progression of damage in the HI+PBS group after 60 days, with changes in the contralateral hemisphere in the form of ventriculomegaly and thinning of the cortex. Subsequent analysis showed a significantly greater lesion volume of 1133±358 mm^3^ in the HI+PBS group when compared with the MSC-EV-treated group, where the lesion volume was 538±151 mm^3^ (p = 0.0036, Figure 4B).

Using the limb-placing test, we found that HI injury of the neonatal brain caused sensorimotor dysfunction of the fore- and hindlimbs. On day 40 and 60 after HI injury, we observed higher neurological status scores in the HI+MSC-EV group (9.2±0.2, p<0.05; and 8.8±0.2 points, p<0.05, respectively) compared with the HI+PBS group, with 4.3±0.3 and 5.5±0.3 points, respectively (Figure 4C). Measurements using the cylinder test on the 60^th^ day after HI injury showed notable asymmetry in the use of the forelimbs. In the TBI+PBS group, the frequency of contralateral forelimb use was 40%±1.7, while MSC-EV treated rats showed significantly greater use of the contralateral forepaws (47%±1.8, p <0.05; Figure 4D).

### MSC-EV protect neurons from injury caused by oxygen-glucose deprivation (OGD) through a decrease in Ca^2+^ overload

As described above, using the HI and TBI models of acute brain injury *in vivo* in newborn and adult rats, respectively, we revealed that uptake of MSC-EV had a pronounced neuroprotective effect, resulting in a smaller volume of damage and sensorimotor functions closer to those of uninjured animals. To decipher the mechanisms of the neuroprotective action of MSC-EV, we ran studies using the OGD cell model, which activates pathogenetic mechanisms similar to those occurring during brain ischemia/reperfusion 57.

**Figure 5:**
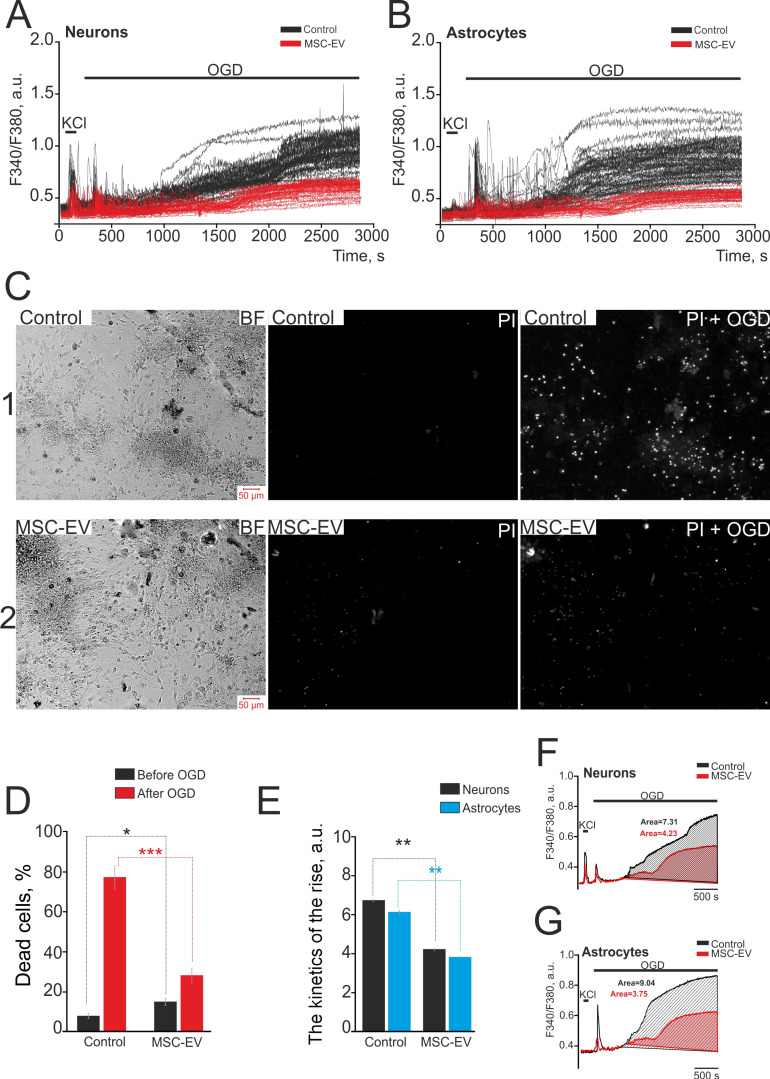
** Neuroprotective effect of 24-h incubation of hippocampal cells with MSC-EV in OGD model. (A)** Set of Ca^2+^ signals from neurons during 40-min OGD in the control (black curves) and after 24h incubation with MSC-EV (red curves). **(B)** Set of Ca^2+^ signals from astrocytes during 40-min OGD in the control (black curves) and after 24h incubation with MSC-EV (red curves). Ca^2+^ signals from single cells are represented using the same cell culture. **(C)** Images of the hippocampal cell culture in transmitted light (BF) and the propidium iodide fluorescence before (PI) and after 40-min OGD (PI+OGD) are given. The white dots represent the nuclei of necrotic cells stained by PI. **(D)** The percentage of PI-stained (necrotic) hippocampal cells before and after OGD in the control group and in cells subjected to 24 h incubation with MSC-EV. **(E)** The effect of MSC-EV on the rate of increase in [Ca^2+^]_i_ in the second phase of the ischemic response of neurons (black columns) and astrocytes (blue columns). **(F, G)** The area under the curve in the second phase of the ischemic response of neurons (F) and astrocytes (G) in the control group and in cells exposed to MSC-EV, reflecting the calcium load of the cells. The differences are statistically significant at *p≤0.05; ** p ≤ 0.01 and ***p ≤ 0.001. Short-term applications of 35 mM KCl and 10 µM ATP were used to detect neurons and astrocytes, respectively. Shown are the means ± SEM for the 2 technical replicates per 3 independent cell cultures. More than 1000 cells were analyzed to build diagrams on panels D and E.

MSC-EV were added to the culture medium of hippocampal neuroglial cultures at a concentration of 2.4×10^8^ particles/mL 24 hours before OGD induction. In neurons (Figure 5A) and astrocytes (Figure 5B) without pre-incubation with MSC-EV (control, black curves), OGD causes the generation of two-phase Ca^2+^ signals. The generation of the first (reversible) Ca^2+^ signal on OGD in neurons occurs on average 1-1.5 min after exposure to the ischemic medium, and the beginning of the phase of global increase in [Ca^2+^]_i_ is observed after 9-12 min. The rate of increase in [Ca^2+^]_i_ during its global rise in control neurons was 6.7±0.03 a.u. (Figure 5E), and the average area under the curve reflecting the Ca^2+^ load in cells was 7.31 a.u. (Figure 5F, black shaded area). In astrocytes of the control group (Figure 5B, black curves), OGD caused the generation of reversible Ca^2+^ signals and the appearance of a global increase in [Ca^2+^]_i_ simultaneously with neurons, but the rate of increase of Ca^2+^ ions in the cytosol in astrocytes during global growth was lower (6.1±0.09 a.u., Figure 5E). On the other hand, the calcium load (the area under the curve) in astrocytes during global growth was 9.04 a.u. (Figure 5G, black shaded area), which is 23.6% higher compared to neurons. In the control, after 40 min of OGD, 78±11% of all cells in the field of view of the microscope were dead (Figure 5C, row 1, marked as PI+OGD), while 7±3% of cells were necrotic before ischemia (Figure 5C, row 1, marked as PI); these data are summarized in Figure 5D.

Neurons (Figure 5A, red curves) and astrocytes (Figure 5B, red curves) in cell culture exposed to MSC-EV, like control cells, also respond to OGD by generating two-phase Ca^2+^ responses, but the amplitude of Ca^2+^ signals during the first phase of the OGD-induced response in neurons from the MSC-EV group was 21±5% smaller, and was 69±14% smaller in astrocytes compared with the control. Moreover, the rate of Ca^2+^ increase in the cytosol of neurons during the second phase of the Ca^2+^ response to OGD (global increase in [Ca^2+^]_i_) was 4.2±0.04 a.u., lower by more than a third than in controls (Figure 5E). The calcium load on neurons during the global increase of Ca^2+^ in the cytosol during OGD, as measured by the area under the curve, was 4.23 a.u. (Figure 5F, red shaded area), which is 42.1% lower compared to the control. In astrocytes after incubation with MSC-EV, the rate of increase in [Ca^2+^]_i_ during the global growth phase was smaller by about a third than in controls (3.8±0.04 a.u., Figure 5E), and the area under the curve was less than half that for controls (Figure 5G, red shaded area), which indicates a decrease in the calcium load in challenged astrocytes.

As to cell death, in cultures exposed to MSC-EV, a slightly higher number of necrotic cells, 14±5%, was observed before OGD (Figure 5C-row 2, marked as PI; Figure 5D), and was significantly higher than in the control group (p=0.016). However, after 40-min OGD, necrotic cell death was observed in only 28±7% of cells (Figure 5C-row 2, shown as PI+OGD; Figure 5D), which was 64.1% less when compared to the control (***, p≤0.001).

### MSC-EV inhibit hippocampal cell apoptosis in hyperammonemia

**Figure 6:**
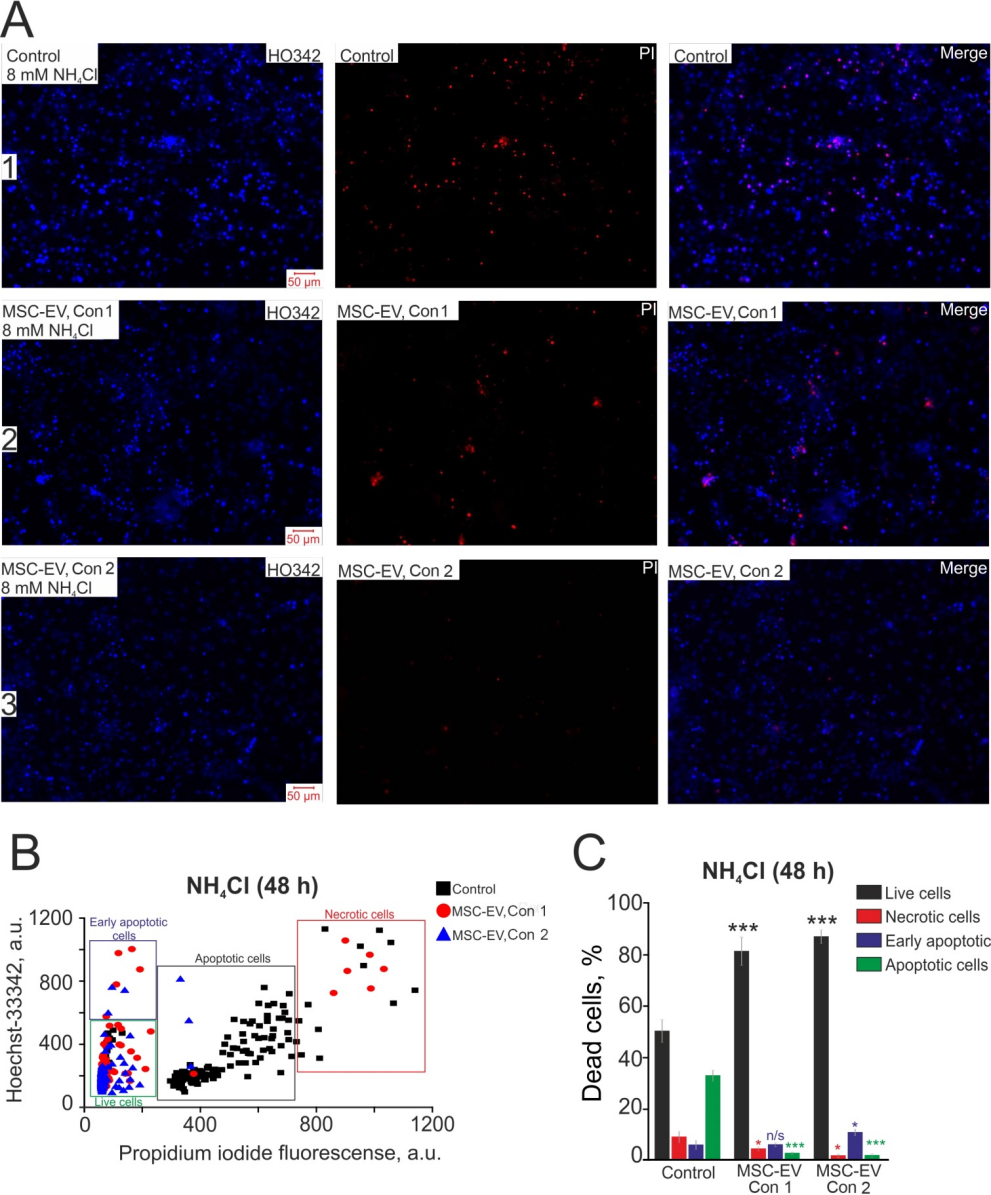
** Antiapoptotic effect of various concentrations of MSC-EV in an *in vitro* model of hyperammonemia. (A)** Simultaneous cell staining with vital DNA dye Hoechst 33342 (HO343, blue) and nonvital DNA dye propidium iodide (PI, red) 48 h after exposure to 8 mM NH_4_Cl. Row 1, control cells; row 2, cells with the addition of MSC-EV at 2.4×10^8^ particles/ml (Con 1); row 3-cells after addition of MSC-EV at 1.6×10^8^ particles/ml (Con 2). **(B)** The scatter plot shows the viability of hippocampal cells. The y-axis represents the intensity of Hoechst 33342 fluorescence. The x-axis shows the intensity of propidium iodide fluorescence in hippocampal cells. Cells were classified based on the intensity of propidium iodide and Hoechst 33342 staining 48 h after hyperammonemia in various experimental groups. **(C)** The effect of MSC-EV on the mode of cell death (necrotic or apoptotic) after 48-h exposure to hyperammonemia. The fraction of live cells (black bar) and cells in which the processes of early apoptosis (purple bar), apoptosis (green bar) and necrosis (red bar) were observed is shown. Shown are the means ± SEM for the 3 technical replicates per 3 independent cell cultures. More than 1000 cells were analyzed to build diagrams on panel C.

Additionally, we tested the efficiency of MSC-EV in protecting against neonatal-onset hyperammonemic encephalopathy. This pathology is quite common in newborns as transient hyperammonemia of the preterm infant 58 or undiagnosed inherited diseases, for example, urea-cycle disorders, or liver pathologies that cause a neurological impairment leading to an acute life-threatening condition 59. After 48 hours of incubation with 8 mM NH_4_Cl in control cultures, late stages of apoptosis were recorded for 37±11% of cells, early apoptosis for 8±5% and necrosis for 12±7%, while 49±7% of cells remained viable (Figure 6A-row 1; Figure 6B-black squares; Figure 6C-Control). In cultures to which MSC-EV were added at a concentration of 2.4×10^8^ particles/mL, after 48 h of exposure, necrosis-type death occurred in 6±3% of cells, and the initial and late stages of apoptosis were recorded in 10±5% and 4±1% of cells, respectively (Figure 6A, row 2; Figure 6B, red circles; Figure 6C, MSC-EV Con 1). Applying a lower concentration of MSC-EV of 1.6×10^8^ particles/mL also had an antiapoptotic effect on hippocampal cells under hyperammonemia: 12±5% and 3±2% of cells were recorded at the early and late stages of apoptosis, respectively, necrotic death was observed in 2±2% of cells, and a larger (83±11%) number of cells remained viable (Figure 6A, row 3; Figure 6B; blue triangles; Figure 6C, MSC-EV Con 2). Thus, the mechanism of the protective effect of MSC-EV in hyperammonemia must involve signaling pathways responsible for inhibiting apoptosis and reducing the levels of necrotic death.

### MSC-EV accelerate the growth of neurites during the development of the hippocampal neuroglial network *in vitro*

**Figure 7:**
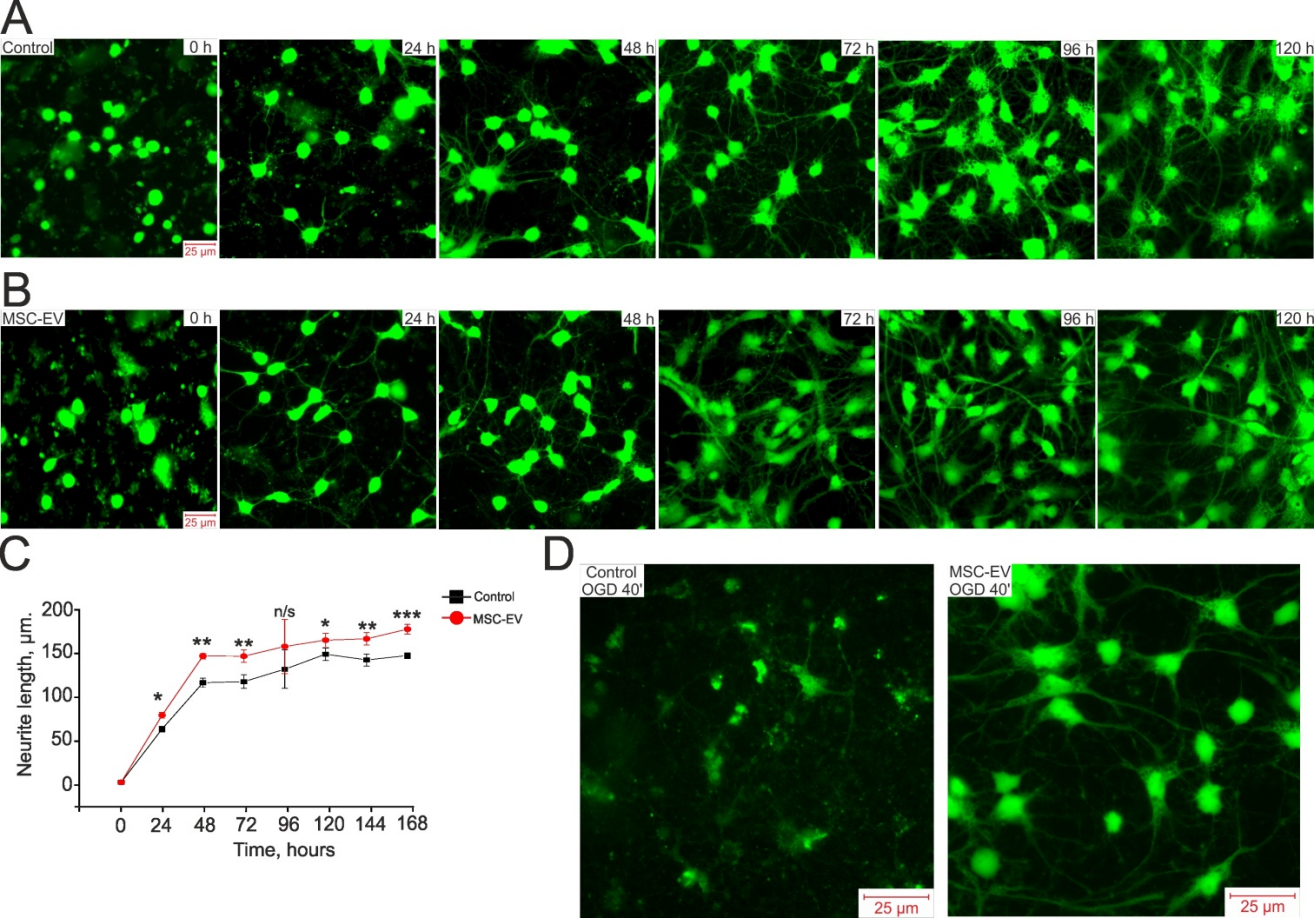
** The effect of MSC-EV on the development of hippocampal cell cultures and the growth of neurites. (A, B)** Images of control cells (A, without the addition of MSC-EV) and cultures with the addition of MSC-EV (B) obtained by fluorescence microscopy. The cells were loaded with the Calcein-AM probe. The time of adding MSC-EV vesicles is shown as 0 h (4 h after cell attachment). Columns show images of cells every 24 h of cultivation for 7 days in culture. **(C)** Changes in the average length of neurites in cultures with and without exposure to MSC-EV. **(D)** Images of Calcein-loaded control and MSC-EV-exposed cells (incubated for 24 h with 2.4×10^8^ particles/mL of MSC-EV) after 40 min of OGD. The differences are statistically significant at *p < 0.05; **p < 0.01 and *** p ≤ 0.001. Statistical significance was assessed using one-way ANOVA followed by the Tukey-Kramer test. Two cell cultures were analyzed for each time point for control and treated conditions.

Since MSC-EV demonstrated pronounced neuroprotective effects and long-term restoration of sensorimotor function after TBI and HI, we suggested that MSC-EV might influence the development of neuroplasticity, which can be realized by a change in neuronal sprouting. The development of the neuroglial network and neuronal sprouting after exposure to MSC-EV was evaluated during 7 days of cell cultivation. After 4 h of cell cultivation, MSC-EV were added to one part of the cell culture in the amount of 1.6×10^8^ particles/mL and the culture medium containing vesicles was not changed during the entire experiment. After the vesicles were added, the cells were loaded with Calcein-AM, which stains both the bodies and neurites of living cells, and fluorescence was recorded. Figure 7A and Figure 7B show images of a control hippocampal cell culture and a culture treated with MSC-EV acquired daily during cultivation. Based on the images (Figure 7A and B, at 0 h) and the measured length of neurites (Figure 7C), both cell cultures were identical at time 0 h. However, after 24 h of cultivation, the length of neurites in the group exposed to MSC-EV was significantly greater than in the control culture (Figure 7B and 7C; * p=0.018). By 48 h of cultivation, the length of neurites in the MSC-EV group was on average 36% greater than in the control (Figure 7B and 7C; ** p=0.0014). On the 4th day of cultivation, the difference in neurite length between the groups was not significant (n/s, p=0.11) due to the large variability in the length of neurites in the MSC-EV group, but the trend of longer length persisted until the 7^th^ day of cultivation, after which the network of neurites in both groups reached a density no longer allowing quantitative processing of data.

We also evaluated the state of neurites after exposure of neuroglial cultures to OGD and the effect of MSC-EV on the preservation of neurites. Figure 7D presents images of Calcein-labeled hippocampal cells after 40 min of OGD. One group of cells was incubated with MSC-EV for 24 h before Calcein loading and OGD while the control group received no MSC-EV. Since Calcein stains only intact parts of the cell, it can be seen that most of the cells are damaged in the control (blurred green profiles) and there is no network of neurites, whereas in a cell culture incubated with vesicles, not only are live cells recorded, but the network of cell neurites is largely preserved.

Thus, MSC-EV stimulates the formation of the neuroglial network and the growth of neurites during culture maturation and contributes to the preservation of the neurite network after OGD. These observations may be of great importance for understanding the protective mechanisms triggered by MSC-EV.

### MSC-EV induce Ca^2+^ oscillations in hippocampal astrocytes without activating vesicles' secretory mechanisms

**Figure 8:**
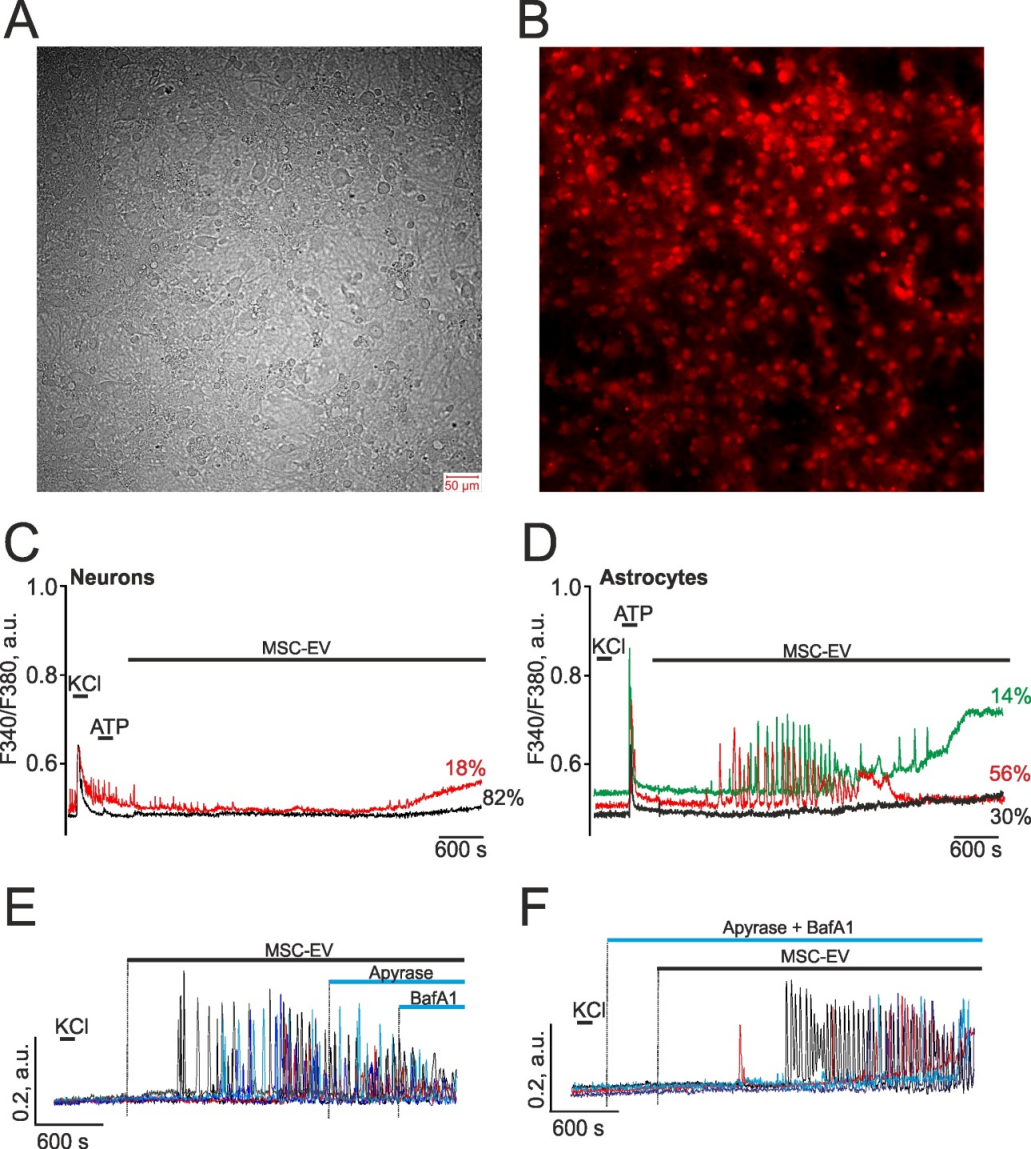
** Exposure to MSC-EV causes the generation of Ca^2+^ oscillations in hippocampal astrocytes.** The role of vesicular ATP secretion. **(A, B)** Hippocampal cell culture on the 14^th^ day of cultivation loaded with a calcium-sensitive probe Fura-2 with recording of transmitted light (A) and of the signal in the 380 nm channel (B). **(C)** Ca^2+^ responses of hippocampal neurons to a short-term addition of 35 mM KCl and demonstrating the absence of signals in response to the addition of MSC-EV at a concentration of 2.4×10^8^ particles/mL. The red curve represents the cumulative signal from neurons undergoing spontaneous Ca^2+^ oscillations while the black curve is a cumulative signal from the population of neurons without spontaneous activity. The averaged signals from several dozen neurons for each curve are presented. **(D)** Ca^2+^ signals from hippocampal astrocytes in response to a short-term addition of 10 µM ATP and MSC-EV at a concentration of 2.4×10^8^ particles/mL. The averaged Ca^2+^ signals obtained from various astrocyte populations are presented. Ca^2+^ oscillations followed by a rise of [Ca^2+^]_i_ stationary level were reсorded in 14% of astrocytes (green curve). In 56% of astrocytes, Ca^2+^ oscillations occured without changing the basal level of [Ca^2+^]_i_ (red curve). Finally, in 30% of astrocytes, [Ca^2+^]_i_ did not respond to the MSC-EV exposure (black curve). The number of astrocytes corresponding to each mode of Ca2+ oscillations is indicated as a percentage. **(E)** MSC-EV-induced Ca^2+^ oscillations of hippocampal astrocytes are not suppressed by the application of 35 units per ml of the ATP-degrading enzyme apyrase and 1 µM of Bafilomycin A1 (BafA1), an inhibitor of V-ATPase. **(F)** Pre-incubation of hippocampal astrocytes with apyrase and BafA1 did not prevent the generation of Ca^2+^ oscillations caused by MSC-EV. Responses of individual astrocytes are presented in panels (E) and (F). Representative Ca^2+^ signals typical for most cells in culture are shown. The experiments were performed in the 4 technical replicates per 3 independent cell cultures.

Since the activation of apoptosis, necrosis, and neurite growth is regulated by calcium ions 60-62, we analyzed the effect of MSC vesicles on the dynamics of cytosolic calcium in hippocampal neurons and astrocytes after 14 days in culture, when they are matured, having already formed a dense network (Figure 8A,B), and the calcium-transporting and receptor systems of these cells have been sufficiently formed. The addition of vesicles at a concentration of 2.4×10^8^ particles/mL to hippocampal cell cultures loaded with the calcium-sensitive probe Fura-2 did not cause Ca^2+^ signals in any neuronal cells during the recording time of 90 min (Figure 8C) or more. However, in a majority of astrocytes, addition of MSC-EV led to the generation of non-synchronous Ca^2+^ oscillations of different frequencies, which occurred on average 10±8 min after exposure to EV (Figure 8D). About 30±11% of astrocytes did not respond to the addition of MSC-EV (Figure 8D, black curve), while in 56±8% of astrocytes, gradually attenuating Ca^2+^ oscillations occurred without changes in the basal levels of [Ca^2+^]_i_ (Figure 8D, red curve), and in 14±9%, attenuating Ca^2+^ oscillations were observed, which were accompanied by an increase in the basal levels of [Ca^2+^]_i_ (Figure 8D, green curve). It should be noted that in network neurons characterized by spontaneous Ca^2+^ oscillations (Figure 8C, red curve), after the addition of MSC-EV, these spontaneous Ca^2+^ oscillations were suppressed and a slow rise in the basal concentration of [Ca^2+^]_i_ occurred, which may be associated with the activation of glial cells and their secretion of ATP and other gliotransmitters 63.

ATP is an essential transmitter secreted by glial cells and mediating cellular communications, including neuroglial interactions 64. In response to the addition of MSC-EV, Ca^2+^ oscillations were recorded in astrocytes with an average frequency of 4±3 oscillations per min and an amplitude of 0.21±0.06 a.u. The addition of the ATP-degrading enzyme apyrase did not suppress the Ca^2+^ oscillations in astrocytes induced by MSC-EV (Figure 8E), but led to a decrease in frequency to 3±2 oscillations per min and the averaged amplitude of oscillations to 0.08±0.04 a.u. Subsequent application of Bafilomycin A1 (BafA1), which blocks ATP secretion through inhibition of V-ATPase in acidic compartments, including ATP-containing vesicles 45, also did not suppress Ca^2+^ oscillations. Pre-incubation of cells with BafA1 and apyrase also did not affect the MSC-EV-induced generation of Ca^2+^ oscillations in astrocytes (Figure 8F), but increased more than 2-fold the lag phase prior to the beginning of oscillations, which on average was 21±7 min, whereas in the control it was 10±8 min.

Thus, MSC-EV caused the generation of Ca^2+^ oscillations in hippocampal astrocytes, and the mechanism of these Ca^2+^ responses did not depend on the vesicular secretion of ATP. However, ATP can play a modulating role or participate in neuroglial interactions, inhibiting spontaneous Ca^2+^ activity of neurons, which, nevertheless, requires further study.

### Activation of the phosphoinositol signaling system in astrocytes by MSC-EV

**Figure 9:**
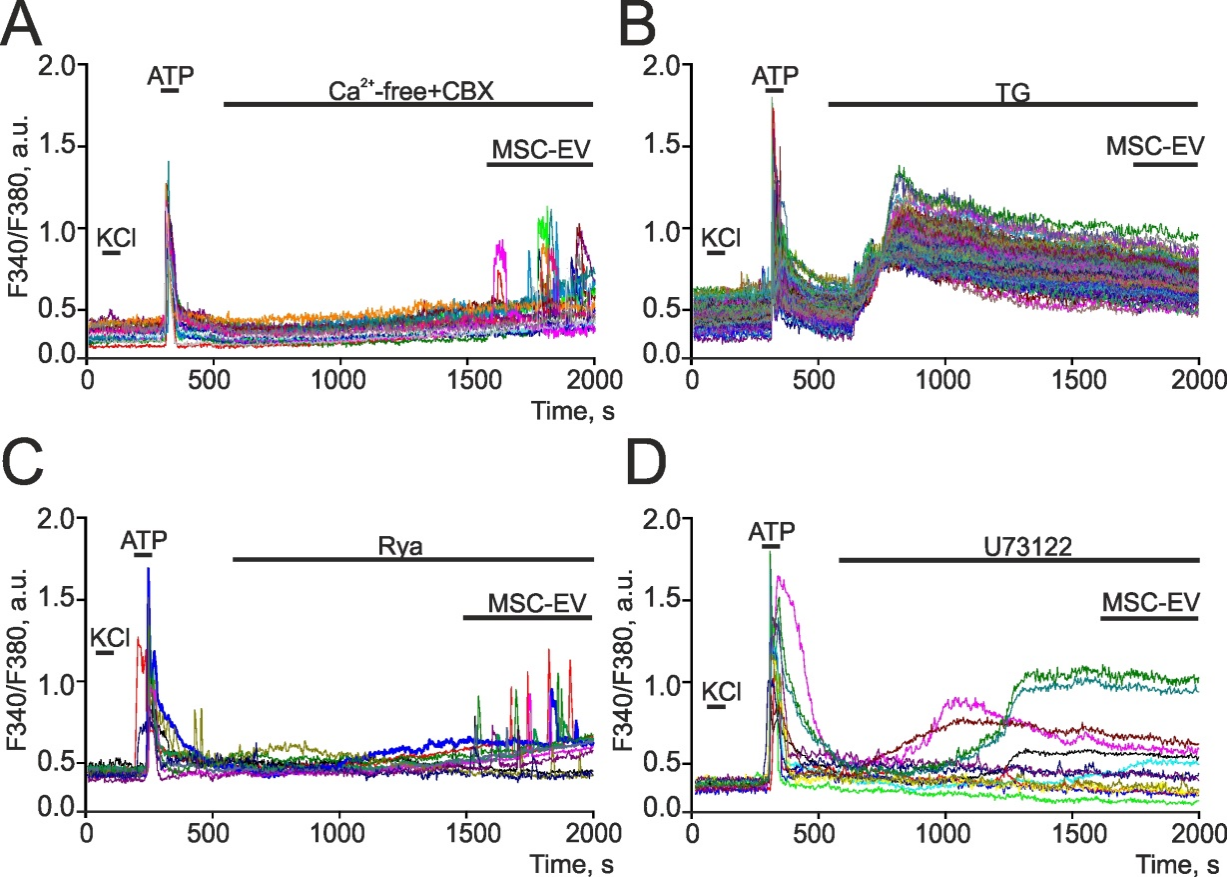
** Identifying the contribution of ryanodine and IP3 receptors to increases in [Ca^2+^]_i_ in hippocampal astrocytes in response to the addition of MSC-EV. (A)** Exposure to MSC-EV at a concentration of 2.4 × 10^8^ particles/mL in a calcium-free medium supplemented with 0.5 mM EGTA and 100 µM carbenoxolone (CBX), an inhibitor of connexin hemichannels. **(B)** Addition of MSC-EV at a concentration of 2.4 × 10^8^ particles/mL after depleting the endoplasmic reticulum Ca^2+^ reserves by adding 1 µM of thapsigargin (TG), an inhibitor of sarco/endoplasmic reticulum Ca-ATPase (SERCA). **(C, D)** Additions of MSC-EV at a concentration of 2.4 × 10^8^ particles/mL with 100 µM ryanodine (Rya), a ryanodine receptor antagonist (C) or 10 µM U73122, an inhibitor of phospholipase C (D). Typical Ca^2+^ signals from astrocytes are presented. Astrocytes were distinguished from neurons by the absence of fast Ca^2+^ signals in response to 35 mM KCl, while exhibiting a high-amplitude Ca^2+^ response to the addition of 10 µM ATP. For this single experiment, representative Ca^2+^ signals are shown. The experiments were performed in the 6 technical replicates per 3 independent cell cultures.

To decipher the mechanisms of action of MSC-EV on neural cells, we analyzed changes in calcium signals in astrocytes exposed to modulators of the phosphoinositol signaling cascade. It is known that eukaryotic cells use both external (from extracellular space) and intracellular (from reserves stored in specialized compartments) sources of calcium ions to generate Ca^2+^ signals. To evaluate the contribution of calcium uptake from the extracellular medium (Figure 9A), MSC-EV were added to astrocytes in a calcium-free medium supplemented with 0.5 mM EGTA, a Ca^2+^ chelator, and 100 µM carbenoxolone (CBX), an inhibitor of connexin hemichannels, the opening of which occurs in a Ca^2+^ containing medium, causing ATP-induced Ca^2+^ signals in astrocytes 65,66. The addition of a calcium-free medium with CBX does not result in Ca^2+^ signals in hippocampal astrocytes, whereas supplementation with MSC-EV led to the generation of transient Ca^2+^ responses or Ca^2+^ oscillations in 47±16% of astrocytes without a significant decrease in amplitude (Figure 9A). The endoplasmic reticulum (ER) is the main Ca^2+^ depot in astrocytes 67. The addition of 1 µM thapsigargin (TG), an inhibitor of sarco/endoplasmic reticulum Ca-ATPase (SERCA), led to the depletion of Ca^2+^ from the ER of astrocytes, as evidenced by an increase in [Ca^2+^]_i_ (Figure 9B), and the further addition of MSC-EV does not induce the generation of Ca^2+^ signals in astrocytes. This, together with the data in Figure 9A, suggests that the mobilization of Ca^2+^ ions from endoplasmic reticulum plays a leading role in the generation of Ca^2+^ signals by astrocytes in response to the addition of vesicles.

The mobilization of Ca^2+^ from ER can occur when ryanodine receptors (RyR) or IP3-receptors (IP3R) are activated. The addition of MSC-EV following incubation with 100 µM Rya, a RyR antagonist 68, does not affect the generation of Ca^2+^ signals by astrocytes, as evidenced by the observation that both transient and oscillatory Ca^2+^ responses were observed in 37±21% of astrocytes under these conditions (Figure 9C). The key enzyme of the phosphoinositide signaling cascade is phospholipase C (PLC), which activity leads to the accumulation of IP3 with subsequent activation of IP3R. Incubation of the hippocampal culture with the inhibitor PLC-U73122 (10 µM), yielded in a complete suppression of the Ca^2+^ responses of astrocytes to MSC-EV (Figure 9D). This implies that Ca^2+^ signals generated in response to MSC-EV exposure were associated with activation of the phosphoinositide Ca^2+^ signaling system of astrocytes.

Thus, the Ca^2+^ signals of astrocytes in response to the addition of MSC-EV originate from mobilization of Ca^2+^ from the ER after IP3R activation, while the ryanodine receptor and uptake of extracellular Ca^2+^ are apparently not involved in this mechanism.

### The protective effect of MSC-EV in OGD conditions is suppressed by PI3K inhibitors

**Figure 10:**
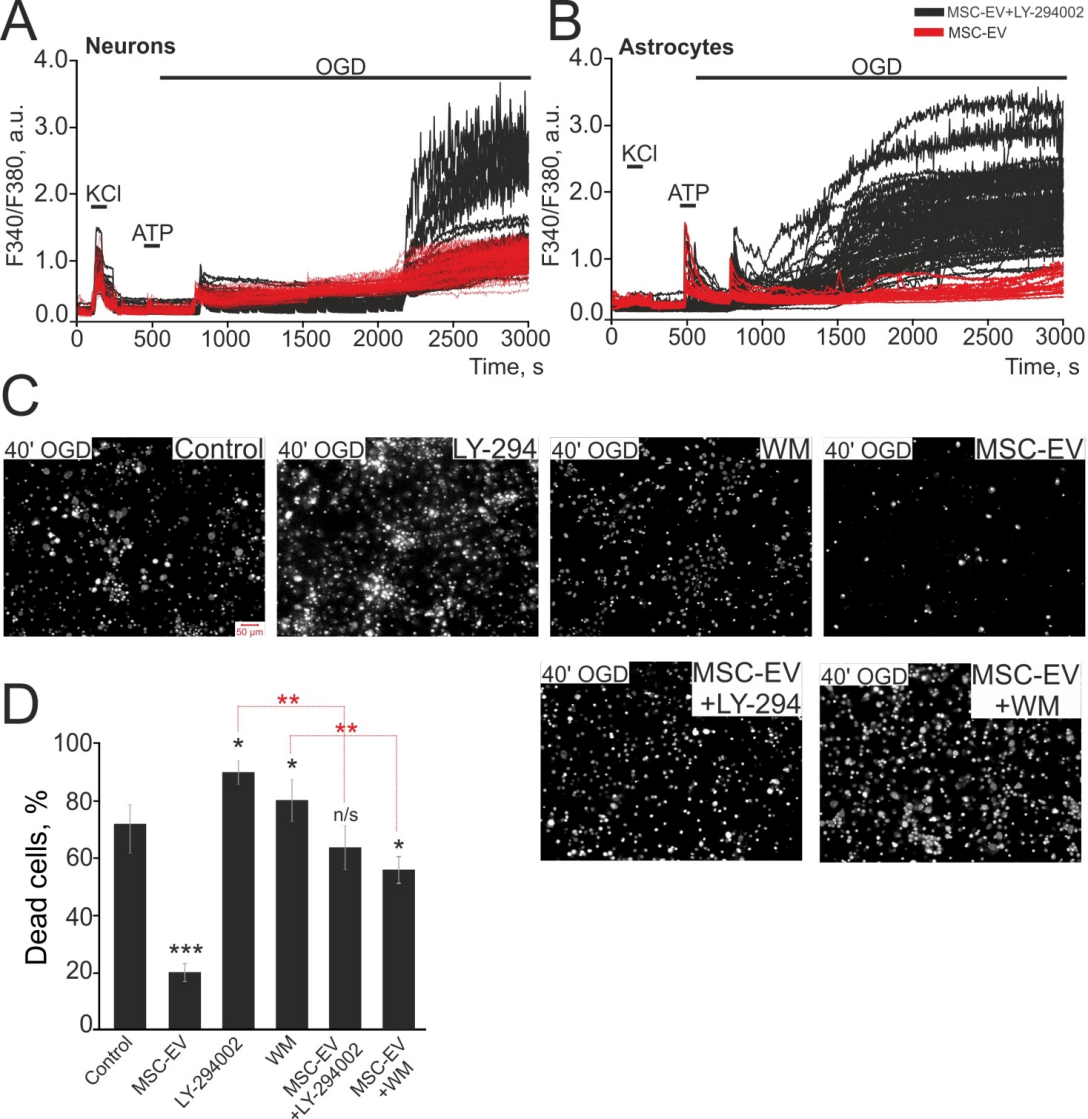
** The effect of inhibitors of PI3K on the protective effect of MSC-EV against OGD. (A, B)** Ca^2+^ responses of neurons (A) and astrocytes (B) during 40 min OGD after 24 h incubation with MSC-EV (red curves) and MSC-EV + 5 µM LY-294002 (selective PI3K inhibitor, black curves). For this single experiment, representative Ca^2+^ signals are shown for (A) and (B). **(C)** Images of hippocampal cell cultures stained with PI after 40 min OGD. The white dots represent the PI-stained nuclei of exclusively necrotic cells. **(D)** The average number of PI-stained (necrotic) hippocampal cells in the control culture and in cultures exposed to 24 h incubation with two inhibitors of PI3K, Wortmannin (WM, 20 µM) and LY-294002 (5 µM), as well as with MSC-EV at a concentration of 2.4 × 10^8^ particles/mL or simultaneous incubation with MSC-EV + 5 µM LY-294002 or MSC-EV + 20 µM Wortmannin (WM). Statistical significance was assessed by one-way ANOVA followed by the Tukey-Kramer test. The differences are statistically significant at *p < 0.05; **p < 0.01 and *** p ≤ 0.001. The differences indicated by black asterisks reflect comparison with the control group (Control). Short-term exposure to 35 mM KCl and 10 µM ATP were used to discriminate neurons and astrocytes, respectively. Shown are the means ± SEM. More than 1000 cells were analyzed to build diagrams on panel D.

To identify the regulatory pathways triggered by MSC-EV, we cultured hippocampal cell cultures with MSC-EV for 24 hours supplemented with two pan-PI3K inhibitors, wortmannin (WM, 20 µM) and selective inhibitor LY-294002 (5 µM), which led to the abolishment of the protective effect of MSC-EV (Figure 10). Incubation of cells with MSC-EV significantly suppressed the OGD-induced global increase in [Ca^2+^]i in neurons (Figure 10A) and completely suppressed it in astrocytes (Figure 10B). This was associated with a smaller fraction of necrotic cells by 68±3% vs control (Figure 10C, D). On the other hand, incubation of cells with MSC-EV + LY-294002 led to a global increase in [Ca^2+^]_i_ during OGD in both neurons (Figure 10A, black curves) and astrocytes (Figure 10B, black curves), which correlates with a greater number of necrotic cells (Figure 10C). OGD after simultaneous incubation of cells with MSC-EV and LY-294002 or MSC-EV and WM leads to an average deaths of up to 63±9% and 57±4% of cells, respectively (Figure 10D). Interestingly, the inhibition of PI3K with LY-294002 or WM without MSC-EV leads to a death rate of 91±4% and 82±11%, respectively, following 40 min of OGD (Figure 10C and Figure 10D), confirming the role of this receptor in protection against OGD.

Thus, phosphoinositide-3-kinase is involved in the mechanism of the protective effect of MSC-EV in OGD as the inhibition of PI3K completely abolishes the suppression of the global increase in [Ca^2+^]_i_ and the inhibition of necrotic processes seen with exposure of hippocampal cells to MSC-EV *in vitro*.

### Proteomic analysis

**Figure 11:**
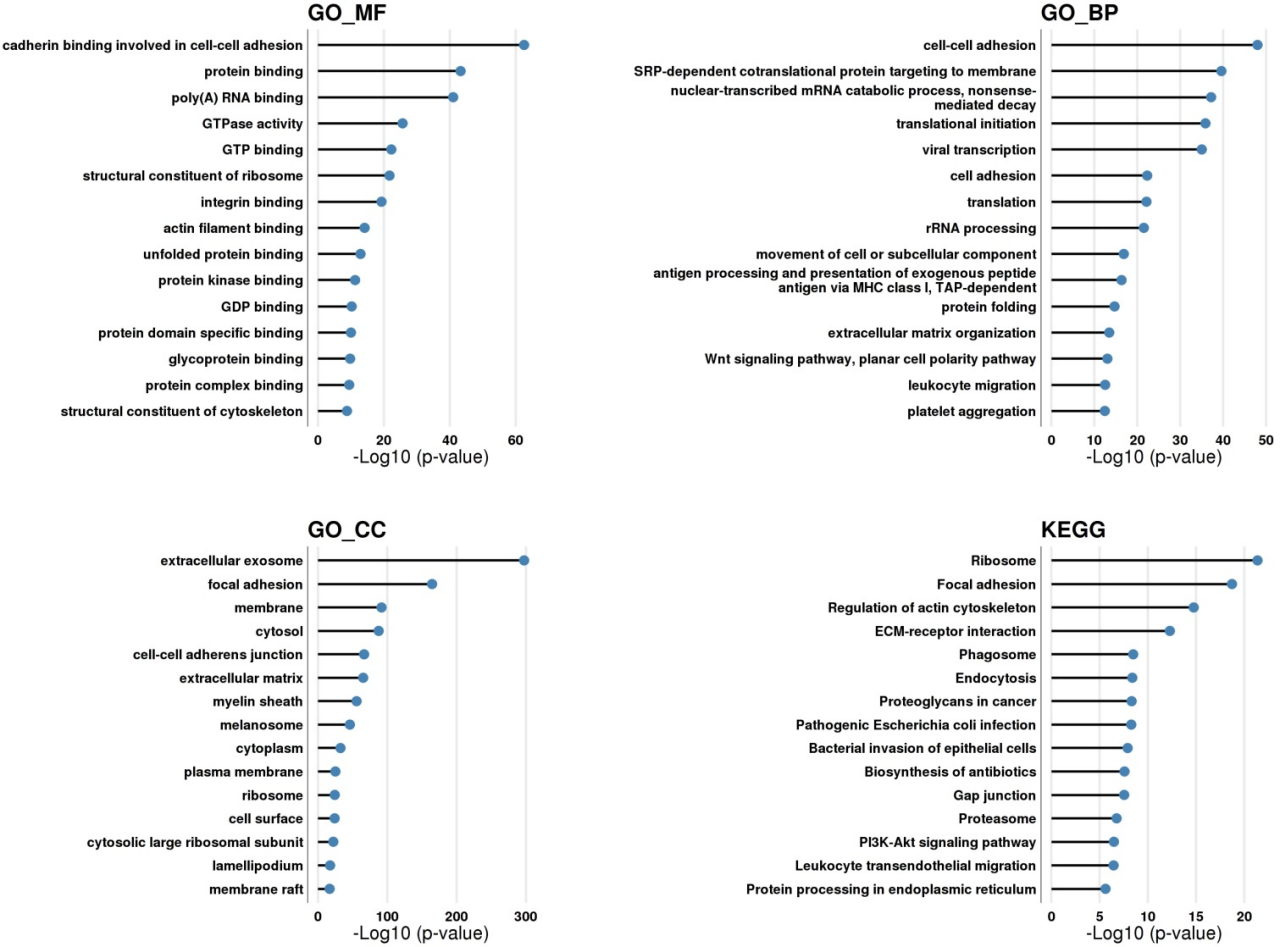
Proteomic analysis of MSC-EV. EV proteins were categorized using KEGG and GO analysis by molecular function, cellular component and biological process in DAVID Bioinformatics Resources, version 6.8.

In order to identify proteins with neuroprotective properties and signaling pathways involved in MSC-EV-mediated neuroprotection, we performed a proteomic analysis of MSC-EV. The proteins of MSC-EV were analyzed using LC-MS/MS, which identified 745 proteins that were further categorized using KEGG and Gene Ontology (GO) analysis by molecular function (MF), cellular component (CC) and biological process (BP) in DAVID Bioinformatics Resources, version 6.8 (Figure 11).

Considering the top enriched GO_CC categories, around 74% of identified MSC-EV proteins were related to the GO_CC category 'extracellular exosome', suggesting that our experiments used a high-purity MSC-EV preparation. Around 6% and 4% of identified proteins are related to GO_CC categories 'ribosome' and 'cytosolic large ribosomal subunit' respectively, suggesting the presence of ribosomes in MSC-EV preparations. Approximately 40%, 39% and 6% of identified proteins are related to GO_CC categories 'membrane', 'plasma membrane' and 'membrane raft' respectively, suggesting that a large portion of identified MSC-EV proteins could be localized in or associated with the membrane of EV's. Around 49% of identified proteins are related to GO_CC categories 'cytosol' and 'cytoplasm', suggesting that half of MSC-EV proteins could be localized within the lumen of EV. Around 26%, 14%, 14% and 11% of identified MSC-EV proteins are related to GO_CC categories 'focal adhesion', 'cell-cell adherent junction', 'extracellular matrix' and 'cell surface' respectively; proteins of these categories are those exposed on the outer surface of EV and are thus the first to communicate and transmit a signal. Around 9.5% of identified MSC-EV proteins are related to GO_CC category 'myelin sheath', suggesting the possible role of MSC-EV in support of nerve fiber integrity and function (Supplementary Table 1 - myelin sheath). Around 7% of identified MSC-EV proteins are related to GO_CC category 'melanosome'; melanosomes may share common features with exosomes due to the same endosomal origin. Finally, about 5% of identified MSC-EV proteins are related to GO_CC category 'lamellipodium', belonging to branched actin filaments that provide force for plasma membrane protrusion during cell migration, which is promoted by the actin-nucleating Arp2/3 complex 69; perhaps cytoskeletal structures within EV could help to maintain the shape of the vesicle, but also could deliver a 'builder' (Arp2/3) to raise cell scaffolds.

Among the top enriched GO_BP categories were those associated with cell adhesion (around 11% of identified proteins), extracellular matrix organization (5%), ribosomes (7%), and cell movement (4%) (with Arp2/3 as one of the major players), which overlap with the top GO_CC categories mentioned above. In addition, around 4% of identified proteins were related to GO_BP category 'leukocyte migration', and around 3% to GO_BP category 'antigen processing and presentation of exogenous peptide antigen via MHC class I, TAP-dependent', suggesting that MSC-EV could play a role in immune-related processes. Around 5% of proteins were assigned to GO_BP category 'protein folding' with such main components as heat shock proteins and TCP1 ring complex (TRiC) (including all its subunits), a eukaryotic molecular chaperone required for the folding of the abundant cytoskeletal proteins actin and tubulin; its role within EV is not yet established, but emerging research suggests its possible therapeutic role in neurodegenerative disorders 70. Around 3.5% of identified proteins belonged to GO_BP category 'Wnt signaling pathway, planar cell polarity pathway'; Wnt5a is a key player in the process of signal transduction and can exert its effects via the PI3K/Akt signaling pathway 71. Around 2% of proteins are related to GO_BP category 'platelet activation', suggesting the role of EV in hemostasis.

Among top GO_MF categories were 'protein binding' (around 75% of identified MSC-EV proteins) such as 'poly(A) RNA binding' (21%), 'cadherin binding involved in cell-cell adhesion' (13.5%), 'GTP binding' (9%), 'GTPase activity' (8%), 'structural constituent of ribosome' (7%), 'protein kinase binding' (7%), 'protein domain specific binding' (4.5%), 'integrin binding' (4.5%), 'protein complex binding' (4%), 'actin filament binding' (4%), 'unfolded protein binding' (4%), 'structural constituent of cytoskeleton' (3%), 'glycoprotein binding' (2.5%) and 'GDP binding' (2%), suggesting a role of MSC-EV in cell adhesion, extracellular matrix organization, signal transduction, cytoskeletal organization, protein folding, and the presence of ribosomes.

Among top enriched KEGG categories were 'ribosome' (6.5% of identified proteins); 'focal adhesion' (8%); 'regulation of actin cytoskeleton' (7%); 'ECM-receptor interaction' (4%); 'phagosome' (4.5%); 'endocytosis' (6%); 'proteoglycans in cancer' (5%); 'pathogenic Escherichia coli infection' (2.5%); 'bacterial invasion of epithelial cells' (3%); 'biosynthesis of antibiotics' (5%); 'gap junction' (3%); 'proteasome' (2%); 'PI3K-Akt signaling pathway' (7%); 'leukocyte transendothelial migration' (3.5%); and 'protein processing in endoplasmic reticulum' (4%), suggesting a role for MSC-EV in adhesion, ECM organization, signal transduction, immune-related processes, the presence of ribosomes and proteasomes.

Among the top revealed KEGG categories is 'PI3K-Akt signaling pathway', experimentally verified to be one of the main pathways by which MSC-EV protect hippocampal neuroglial cells under oxygen-glucose deprivation. According to KEGG, there are 52 proteins in the MSC-EV proteome related to the PI3K-Akt signaling pathway (Supplementary Table 1 - PI3K signaling pathway; Supplementary Figure 1). Depicted routes of signal transduction are: 1) growth factor (GF) → receptor tyrosine kinase (RTK); 2) extracellular matrix (ECM) → integrins (ITGA/ITGB); 3) G-protein coupled receptor (GPCR) → G protein subunit beta/gamma (Gβγ).

For the first route, the following receptors are identified by LC-MS/MS: EPH receptor A2 (EPHA2), epidermal growth factor receptor (EGFR), platelet derived growth factor receptors (PDGFR-α, PDGFR-β); and platelet derived growth factor D (PDGF-DD). For the second route, a range of integrins (ITGA and ITGB) were identified, and the following ECM proteins were revealed by LC/MS: collagens, thrombospondin 1 and 4, laminins, fibronectin, tenascin. For the third route the following receptor was identified: cholinergic receptor muscarinic 2 (CHRM2), and several G proteins.

Moreover, 14-3-3 proteins (YWHAB, YWHAE, YWHAH, YWHAG, YWHAQ, YWHAZ) were identified by proteomic analysis. The 14-3-3 family of proteins mediate signal transduction by binding to phosphoserine-containing proteins and are adapter proteins implicated in the regulation of a large spectrum of both general and specialized signaling pathways.

*In vivo* MSC-EV could contribute to neuroprotection by different routes, stimulating neuro- and gliogenesis, angiogenesis, detoxifying reactive oxygen and nitrogen species, activating anti-apoptotic and anti-necrotic pathways, modifying cell niche through influencing ECM and adhesive properties of cells, triggering specific signaling pathways, through energy supply and through immune-mediated processes, etc. Categories related to these processes were found in the results of DAVID functional annotation 50,51 and Gene Ontology functional annotation performed using The Gene Ontology resource.

The exact mechanisms of the neuroprotective effect of MSC-EV, including the key pathways and proteins responsible for neuroprotection *in vivo*, should be further verified experimentally and are a matter for future research.

### Cytokine analysis of MSC-derived EV

A multiplex approach was used to analyze the cytokine and chemokine content of MSC-EV and there 19 analytes were detected. The highest levels were found for HGF, CXCL1, SCF-b, VEGF-A and MIF, which was the most abundant of these (633 ± 132.43 pg/mL) (Supplementary Figure 2, Supplementary Table 2). Among the identified analytes with concentrations in the range of 50-100 pg/mL, several were among those reported to be significant for brain tissue regeneration, such as VEGF-A 72, CXCL1 73, HGF 74 and MIF 75,76.

## Discussion

Mesenchymal stem cells-derived extracellular vesicles are known to have neuroprotective properties 77. In the clinical aspect, the use of MSC-EV is superior to the parent cells in many reasons including that there are less safety concerns compared to the cells' application 77. Still the EV field is new and much work is needed to confirm safe and productive effects of MSC-EV against neurological disorders and to unravel mechanisms by which MSC-EV exert their therapeutic potential.

There is a large body of data on the participation of calcium ions in inherited and acquired neuropathogenesis. In most cases, it boils down to an undesirable increase in intracellular calcium, which is referred to as cytosolic calcium overload. One of the oldest observations about calcium overload in neurons was its detection in conditions of glutamate toxicity, for example, caused by ischemic challenge 31. As a result of this overload, elevated levels of cytosolic calcium ions lead to mitochondrial Ca^2+^ overload ultimately causing nonspecific permeability of mitochondria and the cell death 30. This imposes a requirement on researchers to unravel the mechanisms of the occurrence of calcium overload and to develop approaches that prevent this overload. This puts the mechanisms of calcium ion transport in the cell and the intracellular processes associated with it at the forefront of the development of a neurotherapeutic strategy.

In this study, we explored the calcium ions-associated intracellular phenomena when examined the neuroprotective potential of MSC-EV, administered intranasally in two *in vivo* models of acute brain injury, specifically, neonatal HI encephalopathy and traumatic brain injury in rats. Intranasal instillation of MSC-EV was used to provide targeted delivery to the damaged brain, simulating the local secretion of EV by MSC 78,79.

In the TBI model, MSC-EV uptake diminished the volume of brain damage, which correlated with significant recovery of sensorimotor functions 30 days after TBI. The late recovery could indicate preferential effects of EV on long-term neuroregeneration by restoring neural connections and neural network necessary for sensorimotor functions.

After neonatal HI injury progressive brain damage was mitigated by EV administration. The volume of brain damage was significantly less in animals treated with MSC-EV compared to untreated animals on day 60 post-injury. In addition, EV promoted recovery of sensorimotor functions in rats 40 and 60 days after HI challenge.

The recovery of sensorimotor functions after MSC-EV administration to animals with HI was less pronounced compared to animals with TBI, probably due to more severe brain damage under HI. Thus, EV attenuated secondary mechanisms of tissue damage and enhanced neuroplasticity, thus recovering neurological function.

To explore the mechanisms of observed MSC-EV neuroprotective potential we employed *in vitro* ischemia model (oxygen-glucose deprivation) using primary hippocampal neurons and astrocytes. Presumably the main adverse factor in OGD is a significant increase of intracellular calcium level that leads to the induction of nonspecific permeability in mitochondria 80 with the subsequent death of astrocyte or neuron. We have shown that MSC-EV protected neurons from injury caused by OGD through a decrease in cytosolic calcium overload.

Thus, addition of MSC-EV to neuroglial culture could influence/modulate calcium fluxes within the cell. Indeed, under normal condition addition of MSC-EV to hippocampal cell cultures induced calcium oscillations in astrocytes, but not in neurons. During those MSC-EV-induced oscillations Ca^2+^ was released from the endoplasmic reticulum with the participation of the IP3 receptor that plays an important role in the activation of the protective anti-ischemic cascade. Under OGD conditions, the protective effect of MSC-EV was suppressed by PI3K inhibitors. The observed mechanism is similar in essence to preconditioning, in which a series of short-term and small pulses of Ca^2+^ ions lead to adaptive changes in the biological system, making it resistant to pathological calcium overload. This adaptive mechanism has been well described for ischemic preconditioning 81, however, in this work, for the first time such phenomena are attributed to the action of EV. It is noteworthy that EV-induced Ca^2+^ oscillations were observed only in astrocytes, and we speculate that EV could induce resistance to calcium overload not only in astrocytes themselves, but also in co-cultured neurons. This indicates a close structural and functional interaction of neurons and astroglial cells, in particular in the process of affording general protection of the brain from ischemic damage 82,83. It can be suggested that MSC-EV cause the generation of pro-survival signals in astrocytes, which are transmitted to other cells, including neurons.

Ultimately, we found that by activation of PI3K signaling pathway and prevention the calcium overload, MSC-EV protected astrocytes and neurons from cell death under OGD.

Besides, MSC-EV prevented apoptosis of neuroglial cells during hyperammonemia, another *in vitro* model of acute ischemia. The mechanism of the protective effect of MSC-EV in hyperammonemia must involve signaling pathways responsible for inhibiting apoptosis and reducing the levels of necrotic death.

In addition to the above-described protective effects, our study also demonstrated that EV stimulated sprouting of neurites, which in brain could provide a spreading of nerves into the injured tissue from adjacent tissues, including penumbra. Presumably, this requires activation of both general protein synthesis and associated processes, as well as the synthesis of various cytoskeletal proteins which undermines that EV carry molecules having many targets including protein synthetic machinery. MSC-EV are known to be highly packed with proteins, nucleic acids, lipids, etc. It is assumed that namely proteins rather than miRNAs could play pivotal role in observed MSC-EV effects considering the beneficial potential of the miRNAs or proteins in a therapeutic dose of exosomes to elicit a biologically relevant response 84. Indeed, some experimental data support the idea that the level of miRNA within EV is extremely low and insufficient to provide any significant activity and influence recipient cells 85,86. Based on this assumption, we conducted proteomic analysis to identify possible candidates in MSC-EV that could exert neuroprotective effects seen in *in vitro* and *in vivo* ischemia models.

The results of DAVID and GO functional annotation of identified proteins indicated that MSC-EV contain proteins that could support a wide spectrum of biological activities with angiogenic 87-89, neurogenic, immunomodulation, anti-oxidative 90 and anti-apoptotic properties. Considering the vast number of identified proteins, it's highly probable that the observed neuroprotection is mediated by the whole range of paracrine factors within MSC-EV through several pathways.

All described proteins are certainly essential, but some MSC-EV proteins are likely to be first to trigger a response in target cells. Specifically, proteins on the surface of EV's can bind directly with the surface receptors of the recipient cell and could initiate downstream signaling. We found the PI3K signaling pathway to be one of the main pathways of MSC-EV-mediated protection of hippocampal neuroglial cells under OGD conditions, and this pathway was identified among the top enriched categories in the MSC-EV proteome. The PI3K/AKT signaling pathway is essential for cell survival and neuroprotection 91 and its modulation by MSC-EV has been recently shown 92-94. Moreover, MSC-conditioned medium protected primary cortical neurons from OGD-induced cell death 95 and inhibition of the PI3K/AKT pathway completely abolished this neuroprotective effect.

The limitation of this study is that females, old animals and those with comorbidities were not included in the study. Future studies are needed to examine the neuroprotective potential of EV according to the important factors of sex, age and specific comorbidities (i.e. diabetes).

In this study, we assumed that the multitargeted therapeutic effect of EV is due to protein cargo that may provide neuroprotection, and point to some key mechanisms and molecular targets for such protection. However, the complexity and variability of EV cargo hamper their translation to the clinic due to the lack of standardized protocols for their production and quality control as well as the lack of knowledge about key mechanisms responsible for their therapeutic effects. We suggest that the issue could be solved by a systematic approach, focused on the investigation of protective pathways induced by EV, specifically pathways associated with neuroprotection.

## Supplementary Material

Supplementary figures and tables 2-3.

Supplementary table 1.

## References

[1] Olesen J, Leonardi M (2003). The burden of brain diseases in Europe. Eur J Neurol.

[2] Lopez AD, Mathers CD, Ezzati M, Jamison DT, Murray CJL (2006). Global and regional burden of disease and risk factors, 2001: systematic analysis of population health data. Lancet.

[3] Frost RB, Farrer TJ, Primosch M, Hedges DW (2013). Prevalence of traumatic brain injury in the general adult population: a meta-analysis. Neuroepidemiology.

[4] Faden AI, Stoica B (2007). Neuroprotection: challenges and opportunities. Arch Neurol.

[5] Johnston SC (2006). Translation: case study in failure. Ann Neurol.

[6] Dhir N, Medhi B, Prakash A, Goyal MK, Modi M, Mohindra S (2020). Pre-clinical to Clinical Translational Failures and Current Status of Clinical Trials in Stroke Therapy: A Brief Review. Curr Neuropharmacol.

[7] Uccelli A, Benvenuto F, Laroni A, Giunti D (2011). Neuroprotective features of mesenchymal stem cells. Best Pract Res Clin Haematol.

[8] Monsel A, Zhu YG, Gennai S, Hao Q, Liu J, Lee JW (2014). Cell-based therapy for acute organ injury: preclinical evidence and ongoing clinical trials using mesenchymal stem cells. Anesthesiology.

[9] He J, Liu J, Huang Y, Tang X, Xiao H, Hu Z (2021). Oxidative Stress, Inflammation, and Autophagy: Potential Targets of Mesenchymal Stem Cells-Based Therapies in Ischemic Stroke. Front Neurosci.

[10] Dihné M, Hartung HP, Seitz RJ (2011). Restoring neuronal function after stroke by cell replacement: anatomic and functional considerations. Stroke.

[11] Babenko VA, Silachev DN, Popkov VA, Zorova LD, Pevzner IB, Plotnikov EY (2018). Miro1 Enhances Mitochondria Transfer from Multipotent Mesenchymal Stem Cells (MMSC) to Neural Cells and Improves the Efficacy of Cell Recovery. Molecules.

[12] Baraniak PR, McDevitt TC (2010). Stem cell paracrine actions and tissue regeneration. Regen Med.

[13] Shimada IS, Spees JL (2011). Stem and progenitor cells for neurological repair: minor issues, major hurdles, and exciting opportunities for paracrine-based therapeutics. J Cell Biochem.

[14] Drago D, Cossetti C, Iraci N, Gaude E, Musco G, Bachi A (2013). The stem cell secretome and its role in brain repair. Biochimie.

[15] Bogatcheva NV, Coleman ME (2019). Conditioned Medium of Mesenchymal Stromal Cells: A New Class of Therapeutics. Biochemistry (Mosc).

[16] Keshtkar S, Azarpira N, Ghahremani MH (2018). Mesenchymal stem cell-derived extracellular vesicles: novel frontiers in regenerative medicine. Stem Cell Res Ther.

[17] Galieva LR, James V, Mukhamedshina YO, Rizvanov AA (2019). Therapeutic Potential of Extracellular Vesicles for the Treatment of Nerve Disorders. Front Neurosci.

[18] Raposo G, Stoorvogel W (2013). Extracellular vesicles: exosomes, microvesicles, and friends. J Cell Biol.

[19] van Balkom BWM, Gremmels H, Giebel B, Lim SK (2019). Proteomic Signature of Mesenchymal Stromal Cell-Derived Small Extracellular Vesicles. Proteomics.

[20] Haraszti RA, Miller R, Dubuke ML, Rockwell HE, Coles AH, Sapp E (2019). Serum Deprivation of Mesenchymal Stem Cells Improves Exosome Activity and Alters Lipid and Protein Composition. iScience.

[21] Takov K, He Z, Johnston HE, Timms JF, Guillot PV, Yellon DM (2020). Small extracellular vesicles secreted from human amniotic fluid mesenchymal stromal cells possess cardioprotective and promigratory potential. Basic Res Cardiol.

[22] Gussenhoven R, Klein L, Ophelders DRMG, Habets DHJ, Giebel B, Kramer BW (2019). Annexin A1 as Neuroprotective Determinant for Blood-Brain Barrier Integrity in Neonatal Hypoxic-Ischemic Encephalopathy. Journal of Clinical Medicine.

[23] Pascua-Maestro R, González E, Lillo C, Ganfornina MD, Falcón-Pérez JM, Sanchez D (2018). Extracellular Vesicles Secreted by Astroglial Cells Transport Apolipoprotein D to Neurons and Mediate Neuronal Survival Upon Oxidative Stress. Front Cell Neurosci.

[24] Lai RC, Tan SS, Teh BJ, Sze SK, Arslan F, de Kleijn DP (2012). Proteolytic Potential of the MSC Exosome Proteome: Implications for an Exosome-Mediated Delivery of Therapeutic Proteasome. Int J Proteomics.

[25] Raposo G, Stahl PD (2019). Extracellular vesicles: a new communication paradigm?. Nat Rev Mol Cell Biol.

[26] Denton RM, Rutter GA, Midgley PJ, McCormack JG (1988). Effects of Ca2+ on the activities of the calcium-sensitive dehydrogenases within the mitochondria of mammalian tissues. J Cardiovasc Pharmacol.

[27] Pandya J, Nukala V, Sullivan P (2013). Concentration dependent effect of calcium on brain mitochondrial bioenergetics and oxidative stress parameters. Frontiers in Neuroenergetics.

[28] Gleichmann M, Mattson MP (2011). Neuronal Calcium Homeostasis and Dysregulation. Antioxidants & Redox Signaling.

[29] Brini M, Calì T, Ottolini D, Carafoli E (2014). Neuronal calcium signaling: function and dysfunction. Cell Mol Life Sci.

[30] Isaev NK, Zorov DB, Stelmashook EV, Uzbekov RE, Kozhemyakin MB, Victorov IV (1996). Neurotoxic glutamate treatment of cultured cerebellar granule cells induces Ca2+-dependent collapse of mitochondrial membrane potential and ultrastructural alterations of mitochondria. FEBS Letters.

[31] Abeti R, Abramov AY (2015). Mitochondrial Ca2+ in neurodegenerative disorders. Pharmacological Research.

[32] Papazian I, Kyrargyri V, Evangelidou M, Voulgari-Kokota A, Probert L (2018). Mesenchymal Stem Cell Protection of Neurons against Glutamate Excitotoxicity Involves Reduction of NMDA-Triggered Calcium Responses and Surface GluR1, and Is Partly Mediated by TNF. Int J Mol Sci.

[33] Théry C, Witwer KW, Aikawa E, Alcaraz MJ, Anderson JD, Andriantsitohaina R (2018). Minimal information for studies of extracellular vesicles 2018 (MISEV2018): a position statement of the International Society for Extracellular Vesicles and update of the MISEV2014 guidelines. J Extracell Vesicles.

[34] Franquesa M, Hoogduijn MJ, Ripoll E, Luk F, Salih M, Betjes MGH (2014). Update on Controls for Isolation and Quantification Methodology of Extracellular Vesicles Derived from Adipose Tissue Mesenchymal Stem Cells. Front Immunol.

[35] Feeney DM, Boyeson MG, Linn RT, Murray HM, Dail WG (1981). Responses to cortical injury: I. Methodology and local effects of contusions in the rat. Brain Res.

[36] Isaev NK, Novikova SV, Stelmashook EV, Barskov IV, Silachev DN, Khaspekov LG (2012). Mitochondria-targeted plastoquinone antioxidant SkQR1 decreases trauma-induced neurological deficit in rat. Biochemistry (Mosc).

[37] Rice JE, Vannucci RC, Brierley JB (1981). The influence of immaturity on hypoxic-ischemic brain damage in the rat. Ann Neurol.

[38] Silachev DN, Uchevatkin AA, Pirogov YA, Zorov DB, Isaev NK (2009). Comparative evaluation of two methods for studies of experimental focal ischemia: magnetic resonance tomography and triphenyltetrazoleum detection of brain injuries. Bull Exp Biol Med.

[39] Jolkkonen J, Puurunen K, Rantakömi S, Härkönen A, Haapalinna A, Sivenius J (2000). Behavioral effects of the alpha(2)-adrenoceptor antagonist, atipamezole, after focal cerebral ischemia in rats. Eur J Pharmacol.

[40] Schallert T, Fleming SM, Leasure JL, Tillerson JL, Bland ST (2000). CNS plasticity and assessment of forelimb sensorimotor outcome in unilateral rat models of stroke, cortical ablation, parkinsonism and spinal cord injury. Neuropharmacology.

[41] Tukhovskaya EA, Turovsky EA, Turovskaya MV, Levin SG, Murashev AN, Zinchenko VP (2014). Anti-inflammatory cytokine interleukin-10 increases resistance to brain ischemia through modulation of ischemia-induced intracellular Ca^2+^ response. Neurosci Lett.

[42] Turovskaya MV, Gaidin SG, Mal'tseva VN, Zinchenko VP, Turovsky EA (2019). Taxifolin protects neurons against ischemic injury *in vitro* via the activation of antioxidant systems and signal transduction pathways of GABAergic neurons. Mol Cell Neurosci.

[43] Zinchenko VP, Turovskaya MV, Teplov IYu, Berezhnov AV, Turovsky EA (2016). The role of parvalbumin-containing interneurons in the regulation of spontaneous synchronous activity of brain neurons in culture. BIOPHYSICS.

[44] Schmid I, Uittenbogaart C, Jamieson BD (2007). Live-cell assay for detection of apoptosis by dual-laser flow cytometry using Hoechst 33342 and 7-amino-actinomycin D. Nat Protoc.

[45] Turovskaya MV, Gaidin SG, Vedunova MV, Babaev AA, Turovsky EA (2020). BDNF Overexpression Enhances the Preconditioning Effect of Brief Episodes of Hypoxia, Promoting Survival of GABAergic Neurons. Neurosci Bull.

[46] Kovalchuk SI, Jensen ON, Rogowska-Wrzesinska A (2019). FlashPack: Fast and Simple Preparation of Ultrahigh-performance Capillary Columns for LC-MS. Mol Cell Proteomics.

[47] Tyanova S, Temu T, Cox J (2016). The MaxQuant computational platform for mass spectrometry-based shotgun proteomics. Nat Protoc.

[48] Tyanova S, Temu T, Sinitcyn P, Carlson A, Hein MY, Geiger T (2016). The Perseus computational platform for comprehensive analysis of (prote)omics data. Nat Methods.

[49] Perez-Riverol Y, Csordas A, Bai J, Bernal-Llinares M, Hewapathirana S, Kundu DJ (2019). The PRIDE database and related tools and resources in 2019: improving support for quantification data. Nucleic Acids Res.

[50] Huang DW, Sherman BT, Lempicki RA (2009). Systematic and integrative analysis of large gene lists using DAVID bioinformatics resources. Nat Protoc.

[51] Huang DW, Sherman BT, Lempicki RA (2009). Bioinformatics enrichment tools: paths toward the comprehensive functional analysis of large gene lists. Nucleic Acids Res.

[52] Ashburner M, Ball CA, Blake JA, Botstein D, Butler H, Cherry JM (2000). Gene ontology: tool for the unification of biology. The Gene Ontology Consortium. Nat Genet.

[53] Gene Ontology Consortium (2021). The Gene Ontology resource: enriching a GOld mine. Nucleic Acids Res.

[54] Keerthikumar S, Chisanga D, Ariyaratne D, Al Saffar H, Anand S, Zhao K (2016). ExoCarta: A Web-Based Compendium of Exosomal Cargo. J Mol Biol.

[55] Mathivanan S, Fahner CJ, Reid GE, Simpson RJ (2012). ExoCarta 2012: database of exosomal proteins, RNA and lipids. Nucleic Acids Res.

[56] Mathivanan S, Simpson RJ (2009). ExoCarta: A compendium of exosomal proteins and RNA. Proteomics.

[57] Tasca CI, Dal-Cim T, Cimarosti H (2015). *In vitro* oxygen-glucose deprivation to study ischemic cell death. Methods Mol Biol.

[58] Ballard RA, Vinocur B, Reynolds JW, Wennberg RP, Merritt A, Sweetman L (1978). Transient Hyperammonemia of the Preterm Infant. New England Journal of Medicine.

[59] Savy N, Brossier D, Brunel-Guitton C, Ducharme-Crevier L, Pont-Thibodeau GD, Jouvet P (2018). Acute pediatric hyperammonemia: current diagnosis and management strategies. HMER.

[60] Berridge MJ (1998). Neuronal calcium signaling. Neuron.

[61] Guo J, Lao Y, Chang DC (2009). Calcium and Apoptosis. In: Lajtha A, Mikoshiba K, editors. Handbook of Neurochemistry and Molecular Neurobiology: Neural Signaling Mechanisms. Springer US. Boston, MA.

[62] Turovsky EA, Varlamova EG (2021). Mechanism of Ca2+-Dependent Pro-Apoptotic Action of Selenium Nanoparticles, Mediated by Activation of Cx43 Hemichannels. Biology.

[63] Gaidin SG, Turovskaya MV, Mal'tseva V.n, Zinchenko VP, Turovsky EA, Blinova EV (2019). A Complex Neuroprotective Effect of Alpha-2-Adrenergic Receptor Agonists in a Model of Cerebral Ischemia-Reoxygenation *In vitro*. Biochemistry (moscow) Supplement Series a: Membrane and Cell Biology.

[64] Marina N, Turovsky E, Christie IN, Hosford PS, Hadjihambi A, Korsak A (2018). Brain metabolic sensing and metabolic signaling at the level of an astrocyte. Glia.

[65] Huckstepp RTR, id Bihi R, Eason R, Spyer KM, Dicke N, Willecke K (2010). Connexin hemichannel-mediated CO2-dependent release of ATP in the medulla oblongata contributes to central respiratory chemosensitivity. J Physiol.

[66] Turovsky EA, Braga A, Yu Y, Esteras N, Korsak A, Theparambil SM (2020). Mechanosensory Signaling in Astrocytes. J Neurosci.

[67] Pivneva T, Haas B, Reyes-Haro D, Laube G, Veh RW, Nolte C (2008). Store-operated Ca2+ entry in astrocytes: different spatial arrangement of endoplasmic reticulum explains functional diversity *in vitro* and *in situ*. Cell Calcium.

[68] Fill M, Copello JA (2002). Ryanodine receptor calcium release channels. Physiol Rev.

[69] Mandrekar N, Su B, Habas R (2018). Chapter 4 - Cell Polarity in Morphogenesis—Planar Cell Polarity. In: Michael Conn P, editor. Cell Polarity in Development and Disease. Boston: Academic Press.

[70] Grantham J (2020). The Molecular Chaperone CCT/TRiC: An Essential Component of Proteostasis and a Potential Modulator of Protein Aggregation. Front Genet.

[71] Kumawat K, Gosens R (2016). WNT-5A: signaling and functions in health and disease. Cell Mol Life Sci.

[72] Zachary I (2005). Neuroprotective role of vascular endothelial growth factor: signalling mechanisms, biological function, and therapeutic potential. Neurosignals.

[73] Karim H, Kim SH, Lapato AS, Yasui N, Katzenellenbogen JA, Tiwari-Woodruff SK (2018). Increase in chemokine CXCL1 by ERβ ligand treatment is a key mediator in promoting axon myelination. Proc Natl Acad Sci U S A.

[74] Tang H, Gamdzyk M, Huang L, Gao L, Lenahan C, Kang R (2020). Delayed recanalization after MCAO ameliorates ischemic stroke by inhibiting apoptosis via HGF/c-Met/STAT3/Bcl-2 pathway in rats. Exp Neurol.

[75] Bae SH, Yoo MR, Kim YY, Hong IK, Kim MH, Lee SH (2020). Brain-derived neurotrophic factor mediates macrophage migration inhibitory factor to protect neurons against oxygen-glucose deprivation. Neural Regen Res.

[76] Zis O, Zhang S, Dorovini-Zis K, Wang L, Song W (2015). Hypoxia signaling regulates macrophage migration inhibitory factor (MIF) expression in stroke. Mol Neurobiol.

[77] Reed SL, Escayg A (2021). Extracellular vesicles in the treatment of neurological disorders. Neurobiology of Disease.

[78] Erdő F, Bors LA, Farkas D, Bajza Á, Gizurarson S (2018). Evaluation of intranasal delivery route of drug administration for brain targeting. Brain Res Bull.

[79] Long Q, Upadhya D, Hattiangady B, Kim DK, An SY, Shuai B (2017). Intranasal MSC-derived A1-exosomes ease inflammation, and prevent abnormal neurogenesis and memory dysfunction after status epilepticus. Proc Natl Acad Sci U S A.

[80] Halestrap AP (2006). Calcium, mitochondria and reperfusion injury: a pore way to die. Biochem Soc Trans.

[81] Hao Y, Xin M, Feng L, Wang X, Wang X, Ma D (2020). Review Cerebral Ischemic Tolerance and Preconditioning: Methods, Mechanisms, Clinical Applications, and Challenges. Frontiers in Neurology.

[82] Semyanov A, Verkhratsky A (2022). Inclusive Brain: From Neuronal Doctrine to the Active Milieu. Function.

[83] Silachev DN, Plotnikov EY, Babenko VA, Savchenko ES, Zorova LD, Pevzner IB (2016). Protection of Neurovascular Unit Cells with Lithium Chloride and Sodium Valproate Prevents Brain Damage in Neonatal Ischemia/Hypoxia. Bull Exp Biol Med.

[84] Toh WS, Lai RC, Zhang B, Lim SK (2018). MSC exosome works through a protein-based mechanism of action. Biochem Soc Trans.

[85] Chevillet JR, Kang Q, Ruf IK, Briggs HA, Vojtech LN, Hughes SM (2014). Quantitative and stoichiometric analysis of the microRNA content of exosomes. Proc Natl Acad Sci U S A.

[86] Albanese M, Chen YFA, Hüls C, Gärtner K, Tagawa T, Mejias-Perez E (2021). Micro RNAs are minor constituents of extracellular vesicles and are hardly delivered to target cells. PLoS Genet.

[87] Merino-González C, Zuñiga FA, Escudero C, Ormazabal V, Reyes C, Nova-Lamperti E (2016). Mesenchymal Stem Cell-Derived Extracellular Vesicles Promote Angiogenesis: Potencial Clinical Application. Front Physiol.

[88] Todorova D, Simoncini S, Lacroix R, Sabatier F, Dignat-George F (2017). Extracellular Vesicles in Angiogenesis. Circ Res.

[89] Xia Y, Ling X, Hu G, Zhu Q, Zhang J, Li Q (2020). Small extracellular vesicles secreted by human iPSC-derived MSC enhance angiogenesis through inhibiting STAT3-dependent autophagy in ischemic stroke. Stem Cell Res Ther.

[90] Luo Q, Xian P, Wang T, Wu S, Sun T, Wang W (2021). Antioxidant activity of mesenchymal stem cell-derived extracellular vesicles restores hippocampal neurons following seizure damage. Theranostics.

[91] Khan H, Singh A, Thapa K, Garg N, Grewal AK, Singh TG (2021). Therapeutic modulation of the phosphatidylinositol 3-kinases (PI3K) pathway in cerebral ischemic injury. Brain Res.

[92] Liu X, Li Q, Niu X, Hu B, Chen S, Song W (2017). Exosomes Secreted from Human-Induced Pluripotent Stem Cell-Derived Mesenchymal Stem Cells Prevent Osteonecrosis of the Femoral Head by Promoting Angiogenesis. Int J Biol Sci.

[93] Adamo A, Brandi J, Caligola S, Delfino P, Bazzoni R, Carusone R (2019). Extracellular Vesicles Mediate Mesenchymal Stromal Cell-Dependent Regulation of B Cell PI3K-AKT Signaling Pathway and Actin Cytoskeleton. Front Immunol.

[94] Brennan MÁ, Layrolle P, Mooney DJ (2020). Biomaterials functionalized with MSC secreted extracellular vesicles and soluble factors for tissue regeneration. Adv Funct Mater.

[95] Scheibe F, Klein O, Klose J, Priller J (2012). Mesenchymal stromal cells rescue cortical neurons from apoptotic cell death in an *in vitro* model of cerebral ischemia. Cell Mol Neurobiol.

