# Supplementary material for: Mesenchymal stromal cell-derived extracellular vesicles afford neuroprotection by modulating PI3K/AKT pathway and calcium oscillations
Source: Int J Biol Sci. 2022 Aug 21;18(14):5345–68. doi: 10.7150/ijbs.73747 (PMC9461662; doi:10.7150/ijbs.73747)
Supplement: Supplementary file 1 — Supplementary figures and tables 2-3. [file ijbsv18p5345s1.pdf]

Appendices

Supplementary Figure 1. KEGG_PATHWAY, hsa04151:PI3K-Akt signaling pathway. Proteins identified by proteomic analysis of MSC-EV are highlighted with red asterisks.

Supplementary Figure 2. The heat map analysis of the cytokine levels in MSC-EV.

Supplementary Table 1. The results of proteomic analysis of MSC-EV and bioinformatic analysis of the MSC-EV proteome.

Supplementary Table 2. EV markers identified by proteomic analysis.

Supplementary Table 3. Analysis of cytokines in MSC-EV.

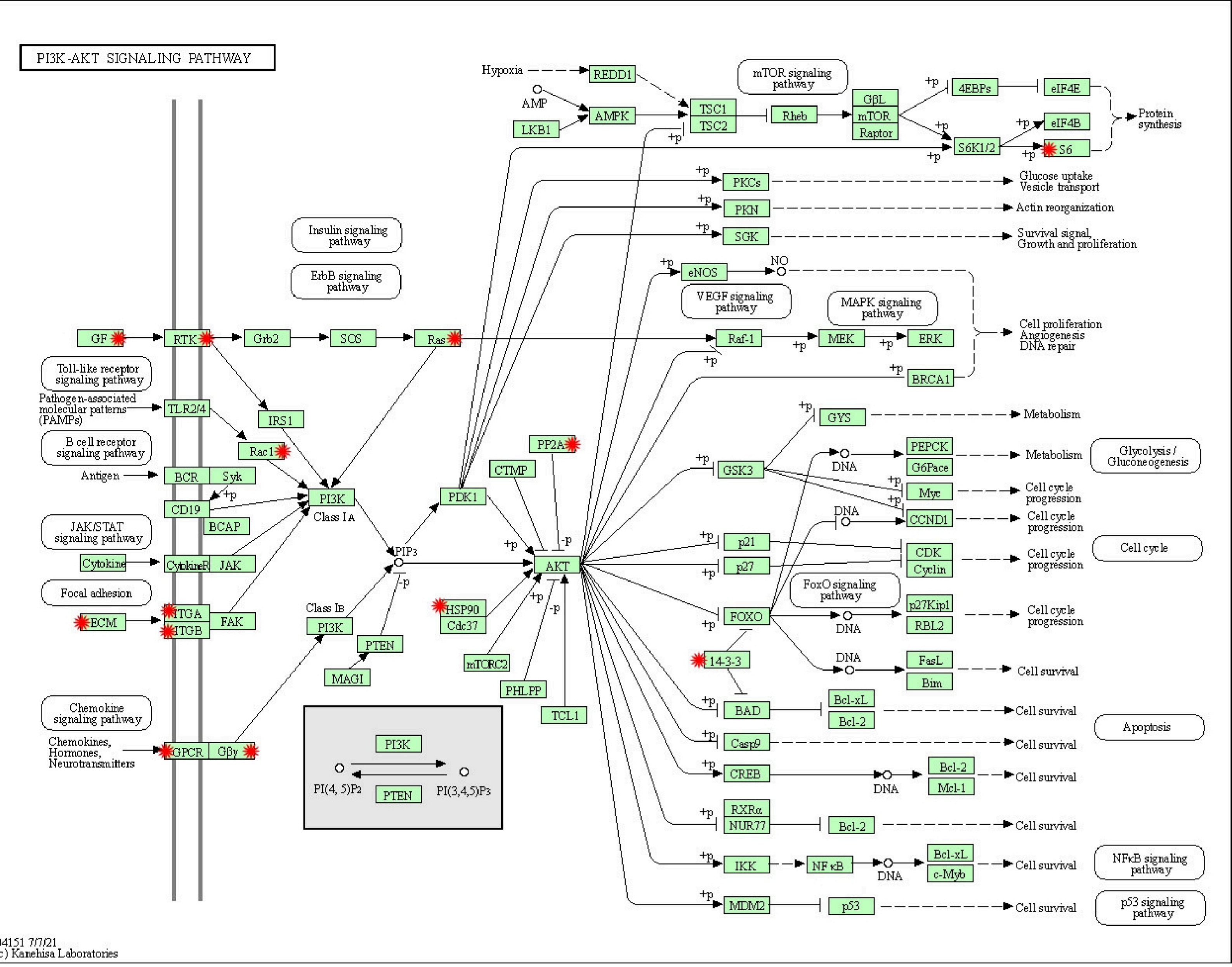
PI3K-AKT SIGNALING PATHWAY
Hypoxia
REDD1
AMP
AMPK
LKB1
TSC1
TSC2
mTOR signaling pathway
Rheb
GβL
mTOR
Raptor
4EBPs
eIF4E
eIF4B
S6K1/2
S6
Protein synthesis
PKCs
Glucose uptake
Vesicle transport
PKN
Actin reorganization
SGK
Survival signal, Growth and proliferation
Insulin signaling pathway
ErbB signaling pathway
eNOS
NO
VEGF signaling pathway
MAPK signaling pathway
GF
RTK
Grb2
SOS
Ras
Raf-1
MEK
ERK
BRCA1
Cell proliferation
Angiogenesis
DNA repair
Toll-like receptor signaling pathway
Pathogen-associated molecular patterns (PAMPs)
TLR2/4
IRS1
Rac1
B cell receptor signaling pathway
Antigen
BCR
Syk
CD19
BCAP
PI3K
Class IA
JAK/STAT signaling pathway
Cytokine
CytokineR
JAK
Focal adhesion
ECM
ITGA
ITGB
FAK
Class IB
PIP3
PTEN
MAGI
Chemokine signaling pathway
Chemokines, Hormones, Neurotransmitters
GPCR
Gβγ
PI(4, 5)P2
PI(3,4,5)P3
PDK1
CTMP
PP2A
AKT
HSP90
Cdc37
mTORC2
PHLPP
TCL1
GYS
Metabolism
GSK3
DNA
PEPCK
G6Pace
Glycolysis / Gluconeogenesis
Myc
Cell cycle progression
CCND1
p21
p27
CDK
Cyclin
Cell cycle
FOXO
FoxO signaling pathway
p27Kip1
RBL2
14-3-3
FasL
Bim
Cell survival
BAD
Bcl-xL
Bcl-2
Apoptosis
Casp9
CREB
Mcl-1
RXRα
NUR77
IKK
NF κB
c-Myb
NFκB signaling pathway
MDM2
p53
p53 signaling pathway
04151 7/7/21
(c) Kanehisa Laboratories

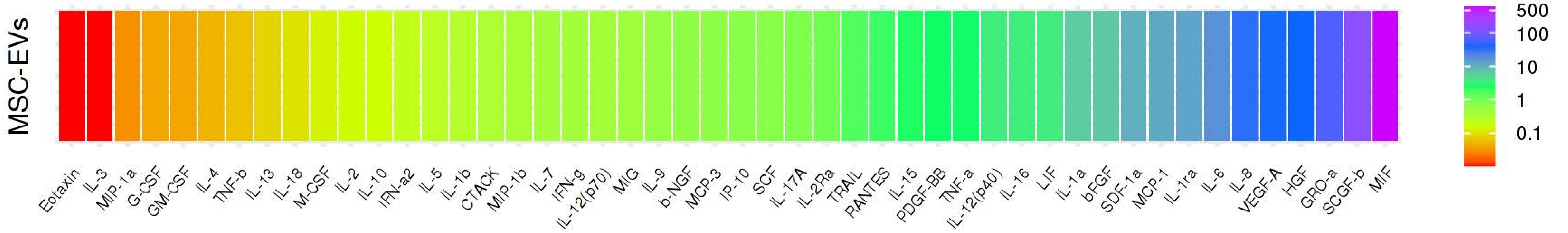
MSC-EVs
Eotaxin
IL-3
MIP-1a
G-CSF
GM-CSF
IL-4
TNF-b
IL-13
IL-18
M-CSF
IL-2
IL-10
IFN-a2
IL-5
IL-1b
CTACK
MIP-1b
IL-7
IFN-g
IL-12(p70)
MIG
IL-9
b-NGF
MCP-3
IP-10
SCF
IL-17A
IL-2Ra
TRAIL
RANTES
IL-15
PDGF-BB
TNF-a
IL-12(p40)
IL-16
LIF
IL-1a
bFGF
SDF-1a
MCP-1
IL-1ra
IL-6
IL-8
VEGF-A
HGF
GRO-a
SCGF-b
MIF
500
100
10
1
0.1

Supplementary Table 2. EV markers identified by proteomic analysis.

**MISEV2018**	**Results of proteomic analysis**
**1- Transmembrane or GPI-anchored proteins associated to plasma membrane and/or endosomes 1a: non-tissue specific**	
Tetraspanins (CD63, CD81, CD82)	CD63, CD81, CD82
other multi-pass membrane proteins (CD47; heterotrimeric G proteins GNA*)	GNA11, GNA13, GNAQ
MHC class I (HLA-A/B/C, H2-K/D/Q)	HLAA, HLAB, HLAC, HLAF, HLAH
Integrins (ITGA*/ITGB*)	ITGB1, ITGB3, ITGAV, ITGA2B, ITGA5, ITGA4, ITGA2, ITGB5, ITGA6, ITGA3, ITGA1, ITGA11
transferrin receptor (TFR2)	-
LAMP1/2	LAMP2
heparan sulfate proteoglycans including syndecans (SDC*)	SDC1
EMMPRIN (BSG)	BSG
ADAM10	ADAM10
GPI-anchored 5'nucleotidase CD73 (NT5E)	NT5E
complement-binding proteins CD55 and CD59	CD55, CD59
sonic hedgehog (SHH)	-
**2 - Cytosolic proteins recovered in EVs 2a: with lipid or membrane protein-binding ability**	
ESCRT-I/II/III (TSG101, CHMP*)	TSG101, CHMP4B
accessory proteins: ALIX (PDCD6IP), VPS4A/B	PDCD6IP
ARRDC1	-
Flotillins-1 and 2 (FLOT1/2)	FLOT1, FLOT2
caveolins (CAV*)	CAV1
EHD*	EHD4, EHD1, EHD3, EHD2
RHOA	-
annexins (ANXA*)	ANXA1, ANXA2, ANXA6, ANXA5, ANXA4, ANXA7, ANXA11
Heat shock proteins HSC70 (HSPA8), and HSP84 (HSP90AB1)	HSPA8, HSP90AB1
ARF6	ARF6
syntenin (SDCBP)	SDCBP
**2 - Cytosolic proteins recovered in EVs 2b: promiscuous incorporation in EVs (and possibly exomeres)**	
Heat shock protein HSP70 (HSPA1A)	HSPA1A
cytoskeleton: actin (ACT*), tubulin (TUB*)	ACTB, ACTA, TUBA1B, TUBA4A, TUBB4B, TUBB3, TUBB8, TUBA1C, TUBB6, TUBB4A, TUBB
enzymes (GAPDH)	GAPDH
**Subtypes of EVs (e.g. large oncosomes, large EVs) and/or pathologic/atypical state 4- Transmembrane, lipid-bound and soluble proteins associated to other intracellular compartments than PM/endosomes**	
**4a: nucleus**	
Histones (HIST1H**)	HIST1H2AJ, HIST1H2BL
Lamin A/C (LMNA)	-
**4b: mitochondria**	
IMMT	-
cytochrome C (CYC1)	-
TOMM20	-
**4c: secretory pathway**	
Calnexin (CANX)	CANX

Grp94 (HSP90B1)	HSP90B1
BIP (HSPA5)	HSPA5
GM130 (GOLGA2)	-
**4d: others (autophagosomes, cytoskeleton…)**	
ATG9A	-
Actinin1/4 (ACTN1/4)	ACTN1, ACTN4
cytokeratin 18 (KRT18)	-
**5- Secreted proteins recovered with EVs**<br>secreted or luminal proteins that can associate with EVs by binding to specific (e.g. growth factor receptors) or to promiscuous (e.g. proteoglycan, lipid) receptors on the EV surface	
**5a: Cytokines and growth factors**	
TGFB1/2	-
IFNG	-
VEGFA	-
FGF1/2	-
PDGF*	PDGFD
EGF	-
interleukins (IL*)	-
**5b: adhesion and extracellular matrix proteins**	
Fibronectin (FN1)	FN1
Collagen (COL**)	COL1A2, COL6A1, COL6A2, COL6A3, COL5A1, COL12A1, COL1A1
MFGE8	MFGE8
galectin3-binding protein (LGALS3BP)	LGALS3BP
CD5L	-
fetuin-A (AHSG)	-
**3- Major components of non-EV co-isolated structures**<br>**All EVs as purity control**	
**3a: lipoproteins (produced by liver, abundant in plasma, serum)**	
Apolipoproteins A1/2 and B APOA1/2, APOB; APOB100; albumin (ALB)	APOB
**3b: protein and protein/nucleic acid aggregates**	
Ribosomal proteins	RPS20, RPS3A, RPL26, RPL15, RPL27, RPS8, RPS15A, RPS16, RPS14, RPS23, RPS18, RPS13, RPS11, RPL7A, RPS4X, RPS6, RPL23, RPS15, RPS25, RPL30, RPL10A, RPL32, RPL11, RPL8, RPS27A, RPL24, RPL19, RPL18A, RPL6, RPL18, RPL36, RPLP2, RPLP0P6, RPSA, RPS2, RPL7, RPL17, RPS3, RPS12, RPL13, RPL10, RPL12, RPL9, RPL4, RPL3, RPL13A, RPS27, RPL27A, RPL5, RPL21, RPL28, RPS9, RPS5, RPL14

Notice. Left column – groups of EVs protein markers, recommended by MISEV2018 [PMID: 30637094]; right column – proteins of MSC-EVs identified by proteomic analysis; '-' – the protein was not identified by current proteomic analysis.

Supplementary Table 3. Analysis of cytokines in MSC-EV.

Cytokine	MSC-EV (pg/mL)
bFGF	6,68 ± 4,83
CTACK	0,35 ± 0,01
Eotaxin	0,01 ± 0,003
G-CSF	0,04 ± 0,03
GM-CSF	0,04 ± 0,03
GRO-a	61,6 ± 50,57
HGF	40,79 ± 32,74
IFN-a2	0,21 ± 0,01
IFN-g	0,44 ± 0,18
IL-1a	6,39 ± 4,82
IL-1b	0,29 ± 0,23
IL-1ra	12,78 ± 1,13
IL-2	0,15 ± 0,09
IL-2Ra	1,08 ± 0,38
IL-3	0,01 ± 0
IL-4	0,05 ± 0,03
IL-5	0,24 ± 0,07
IL-6	17,97 ± 8,32
IL-7	0,43 ± 0,38
IL-8	34,43 ± 21,76
IL-9	0,56 ± 0,45
IL-10	0,16 ± 0,06
IL-12(p40)	3,4 ± 0,14
IL-12(p70)	0,44 ± 0,13
IL-13	0,08 ± 0,03
IL-15	2,34 ± 0,42
IL-16	3,41 ± 1,4
IL-17A	0,86 ± 0,18
IL-18	0,1 ± 0,06
IP-10	0,71 ± 0,45
LIF	3,52 ± 1,86
MCP-1	12,06 ± 6,07
MCP-3	0,66 ± 0,39
M-CSF	0,13 ± 0,04
MIF	633 ± 132,43
MIG	0,48 ± 0,34
MIP-1a	0,03 ± 0,02
MIP-1b	0,37 ± 0,04
b-NGF	0,64 ± 0,54
PDGF-BB	2,46 ± 0,63
RANTES	1,99 ± 0,39
SCF	0,75 ± 0,32
SCGF-b	150,9 ± 80,78
SDF-1a	10,91 ± 10,74
TNF-a	2,6 ± 0,69
TNF-b	0,06 ± 0,01

TRAIL	1,65 ± 1,21
VEGF-A	37,82 ± 21,05
